# Consequences of introgression and gene flow on the genetic structure and diversity of Lima bean (*Phaseolus lunatus* L.) in its Mesoamerican diversity area

**DOI:** 10.7717/peerj.13690

**Published:** 2022-07-05

**Authors:** Mauricio Heredia-Pech, Mariana Chávez-Pesqueira, Matilde M. Ortiz-García, Rubén Humberto Andueza-Noh, María Isabel Chacón-Sánchez, Jaime Martínez-Castillo

**Affiliations:** 1 Unidad de Recursos Naturales, Centro de Investigación Científica de Yucatán, A.C., Mérida, Yucatán, México; 2 División de Estudios de Posgrado e Investigación, Instituto Tecnológico de Conkal, Conkal, Yucatán, México; 3 Departamento de Agronomía, Facultad de Ciencias Agrarias, Universidad Nacional de Colombia, Bogotá, D.C., Colombia

**Keywords:** SNPs markers, Mayan milpa, Yucatan Peninsula, Wild-weedy-crop complexes, Wild-crop introgression

## Abstract

We evaluated the role of gene flow and wild-crop introgression on the structure and genetic diversity of Lima bean (*Phaseolus lunatus*) in the Yucatan Peninsula, an important Mesoamerican diversity area for this crop, using a genotyping-by-sequencing approach (15,168 SNP markers) and two scales. At the local scale, STRUCTURE and NGSEP analyses showed predominantly crop-to-wild introgression, but also evidence of a bidirectional gene flow in the two wild-weedy-crop complexes studied (Itzinté and Dzitnup). The ABBA-BABA tests showed a higher introgression in Itzinté (the older complex) than in Dzitnup (the younger one); at the allelic level, the wild-crop introgression in Itzinté was similar in both directions, in Dzitnup it was higher from crop-to-wild; and at the chromosomal level, introgression in Itzinté was from wild-to-crop, whereas in Dzitnup it occured in the opposite direction. Also, we found *H*_E_ values slightly higher in the domesticated accessions than in the wild ones, in both complexes (Itzinté: wild = 0.31, domesticated = 0.34; Dzinup: wild = 0.27, domesticated = 0.36), but %*P* and *π* estimators were higher in the wild accessions than in the domesticated ones. At a regional scale, STRUCTURE and MIGRATE showed a low gene flow, predominantly from crop-to-wild; and STRUCTURE, Neighbor-Joining and PCoA analyses indicated the existence of two wild groups and one domesticated group, with a marked genetic structure based in the existence of domesticated MI and wild MII gene pools. Also, at the regional scale, we found a higher genetic diversity in the wild accessions than in the domesticated ones, in all estimators used (*e.g.*, *H*_E_ = 0.27 and *H*_E_ = 0.17, respectively). Our results indicate that gene flow and introgression are playing an important role at the local scale, but its consequences on the structure and genetic diversity of the Lima bean are not clearly reflected at the regional scale, where diversity patterns between wild and domesticated populations could be reflecting historical events.

## Introduction

Future climate scenarios foresee an increase in temperature and variability in rainfall, which would have negative consequences for food production worldwide (IPCC, 2014). Global climate change will strongly impact intraspecific diversity (Hoffmann & Sgrò, 2011), which could in turn reduce genetic diversity in natural populations and contribute to a reduction in their viability and ultimately their extinction (Pauls et al., 2013). For domesticated species, two major concerns are the decrease in productivity and the increase in post-harvest losses (Burgarella et al., 2019). Moreover, domesticated species usually bear low levels of genetic diversity compared to their wild relatives, as a result of recurring cycles of selection during their domestication and subsequent improvement (Meyer & Purugganan, 2013). Gene flow (*i.e.,* the exchange of genetic material between different gene pools, whether of the same or different species; Slatkin, 1985) and introgression (*i.e.,* the permanent incorporation of genes from one population to another, recurrent events of backcrosses of the hybrids with the parent and vice versa; Anderson, 1949), represent the immediate primary sources for broadening the genetic base of domesticated species. Recurrent events of gene flow and introgression between domesticated species and their wild relatives can generate the existence of wild-weedy-crop complexes, defining weedy as the individuals resulting from the hybridization between domesticated and wild individuals. These type of complexes can become established over time and generate a wide variety of individuals with admixed ancestry (Ellstrand et al., 2013); such genetic variability could be directly exploited by traditional farmers and/or is potentially useful in breeding programs.

While wild-crop gene flow and introgression can be studied at a regional scale, which allows us to understand the historical genetic consequences of both processes, it is also advisable to study both processes at a local scale, since this allows us to gain knowledge of the immediate genetic consequences and relate them with the agricultural management in the involved agroecosystems (Dzul-Tejero, Coello-Coello & Martínez-Castillo, 2014). The effect that gene flow and introgression can have on populations depends on the degree and direction of gene movement, but their importance in shaping the genetic diversity and structure of domesticated species and their wild relatives is generally recognized (Ellstrand, 2014). Thus, new genetic combinations resulting from wild-to-crop gene flow and introgression play a vital role in the evolution of domesticated species and continue to have a significant effect in increasing the genetic diversity of modern crops (Harlan, 1965; Jarvis & Hodgkin, 1999; Anderson & De Vicente, 2010). In contrast, crop-to-wild gene flow and introgression can lead to a reduction in the genetic diversity of wild relatives, local extinction of wild populations, and the emergence of more aggressive weed varieties (Ellstrand, Prentice & Hancock, 1999).

Lima bean (*Phaseolus lunatus* L.) is one of the five domesticated species of the genus *Phaseolus*, comprising two botanical varieties (Baudet, 1977): *P. lunatus* var. *lunatus* that include all domesticated populations (landraces and improved varieties) and *P. lunatus* var. *silvester* that include the wild populations. This species shows a very wide range of ecological adaptations throughout its natural distribution, from Mexico to Argentina; being considered a promising crop to improve food security under the climate change scenarios predicted for Latin America and other regions of the world where it is grown (Delgado-Salinas & Gama-López, 2015). Lima bean is a self-compatible species with a mixed reproductive system, but is predominantly autogamous (Webster, Lynch & Tucker, 1979), though in some studies high outcrossing rates have been reported (Baudoin et al., 1998; Zoro Bi, Maquet & Baudoin, 2005; Penha et al., 2016). In wild populations of Lima bean, movement of pollen and seeds usually does not exceed 6 m (Hardy et al., 1997; Baudoin et al., 1998), which favor low levels of gene flow and high levels of genetic structuring at relatively small spatial scales (Ouédraogo & Baudoin, 2002; Martínez-Castillo et al., 2007). Wild Lima bean populations show a metapopulation dynamic characterized by extinction-recolonization processes favored by the formation of seed banks with a viability of up to three years (Degreef et al., 2002; Barrantes et al., 2008). The phylogeographic structure of the Lima bean is composed of three large gene pools, both containing wild and domesticated populations: Andean I (AI), that is mainly distributed in the Andes of Ecuador and northern Peru; Mesoamerican I (MI), that only exists in Mexico; and Mesoamerican II (MII) with a distribution that ranges from southern Mexico to South America; though the existence of a fourth gene pool (Andean II) with a distribution in the Andes of central Colombia in the north of South America, has also been indicated (Chacón-Sánchez & Martínez-Castillo, 2017; Garcia et al., 2021).

In the Yucatan Peninsula, Mexico, the MI and MII gene pools of Lima bean converge; to date, the existence of mainly MI domesticated populations, introduced in pre-Columbian times from the area of domestication in central-western Mexico (only one MII domesticated accession has been reported), and local MII wild populations have been reported (Chacón-Sánchez & Martínez-Castillo, 2017). In this region, there is evidence of Lima bean cultivation for at least 1000 years (Kaplan & Kaplan, 1988), and this crop is currently considered the fourth most important species within the traditional agriculture system known as Mayan milpa (Martínez-Castillo et al., 2004). The Mayan milpa is an agricultural system based on periodic cultivation, where the vegetation is cyclically slashed and burned to plant crops in the area during a period of 1–3 years and then left fallow for the next 5–15 years when a new cycle can be initiated (Hernández-Xolocotzi, 1959). Moreover, the Yucatan Peninsula is the region with the highest number of landraces of Lima bean in Mexico (Ballesteros, 1999; Martínez-Castillo et al., 2004), as well as an important region with wild populations that possess high levels of diversity (Martínez-Castillo et al., 2006). For all these reasons, the Yucatan Peninsula is considered a center of genetic diversity for the Lima bean in Mesoamerica, and an ideal natural laboratory to study the role that wild-crop gene flow and introgression may play in the genetic diversity and structure of this species. These two microevolutionary processes may be favored by some characteristics of the Mayan milpa: (a) itinerancy, which favors the physical contact between wild and domesticated populations; (b) fire management, which assists in the regeneration of wild populations from seed banks; (c) manual weeding (as opposed to the use of herbicides, tractors or other agricultural machinery) allows some wild plants to evade removal and thus to reach its reproductive stage along with domesticated plants; (d) production intended mainly for self-consumption, which promotes more relaxed farmer selection criteria that allow the maintenance of weedy seeds; and (e) cultivation of many landraces in the same milpa, which can lead to a wide variety of seeds with different shapes, colors and sizes that can mask the presence of weedy seeds (Chacón-Sánchez et al., 2021).

In the Yucatan Peninsula, Martínez-Castillo et al. (2007) and Dzul-Tejero, Coello-Coello & Martínez-Castillo (2014) assessed wild-crop gene flow and introgression in Lima bean at different spatial scales using microsatellite markers (SSRs- Single Simple Repeats); however, both used a limited genomic sampling. Thanks to the advances in next-generation sequencing (NGS) and genomic markers, such as single nucleotide polymorphisms (SNPs), it is now possible to carry out genome-wide studies that allow more precise determination of genetic diversity within and between populations (Glover et al., 2010; Gärke et al., 2012; Fischer et al., 2017) and determine population genetic structure at a more precise scale (Singh et al., 2013). In the case of gene flow and introgression studies, the use of markers such as SNPs has made it possible to accurately estimate intra and interspecific gene flow, which in turn allows evaluating introgression events, as well as, distinguishing progenitors of recent hybrids, and identifying introgressed chromosome blocks (Twyford & Ennos, 2012).

Given the important role that gene flow and introgression play in the evolutionary dynamics of domesticated species and their wild relatives, the objectives of this study were to determine: (1) the degree and direction of introgression at a local scale in two wild-weedy-crop complexes of Lima bean of different ages, (2) the degree and direction of wild-crop gene flow in Lima bean at a regional scale in the Yucatan Peninsula, and (3) the consequences of these two processes on the structure and genetic diversity of Lima bean in this region of Mexico. To meet these goals, we used 15,168 SNP markers genotyped in 183 accessions and a population genomic approach.

## Survey Methodology

### Study sites and plant material

The study was conducted in the Yucatan Peninsula, Mexico, at a local and regional scale:

(1) Local scale. A specific search for wild-weedy-crop complexes of Lima bean was carried out in 2019; two complexes were found, which were named as Itzinté and Dzitnup based on local place names (from this moment on, the use of these names will only refer to the existence of the complexes, unless otherwise indicated). As species, *P. lunatus* is not in any risk category, all material plant used in this study was collected under scientific permits (SGPA/DGVS/008421/18 and SGPA/DGGFS/712/2913/17) issued by the Secretaría del Medio Ambiente y Recursos Naturales (SEMARNAT-Mexico) to researchers at Centro de Investigación Científica de Yucatán, A. C (CICY).

**Figure 1:**
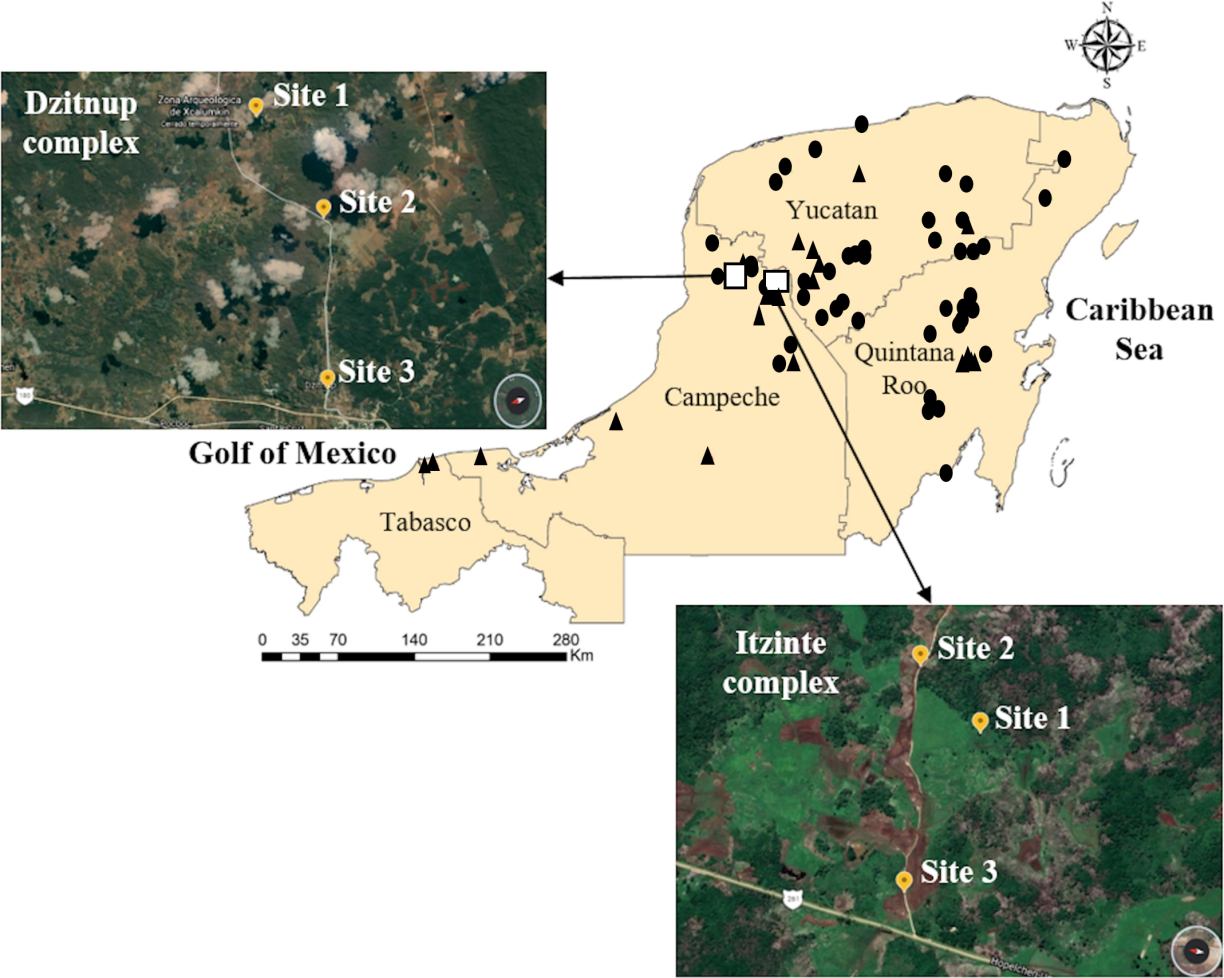
Geographic distribution of wild and domesticated Lima bean accessions collected in the Yucatan Peninsula, Mexico. Domesticated accessions are shown in black circles and wild accessions in black triangles, the white boxes show the collection sites of the two studied wild-weedy-crop complexes: Dzitnup and Itzinté. Source Credit: Landsat/Copernicus INEGI Data SIO, NOAA, U.S. Navy, NGA, GEBCO.

**Figure 2:**
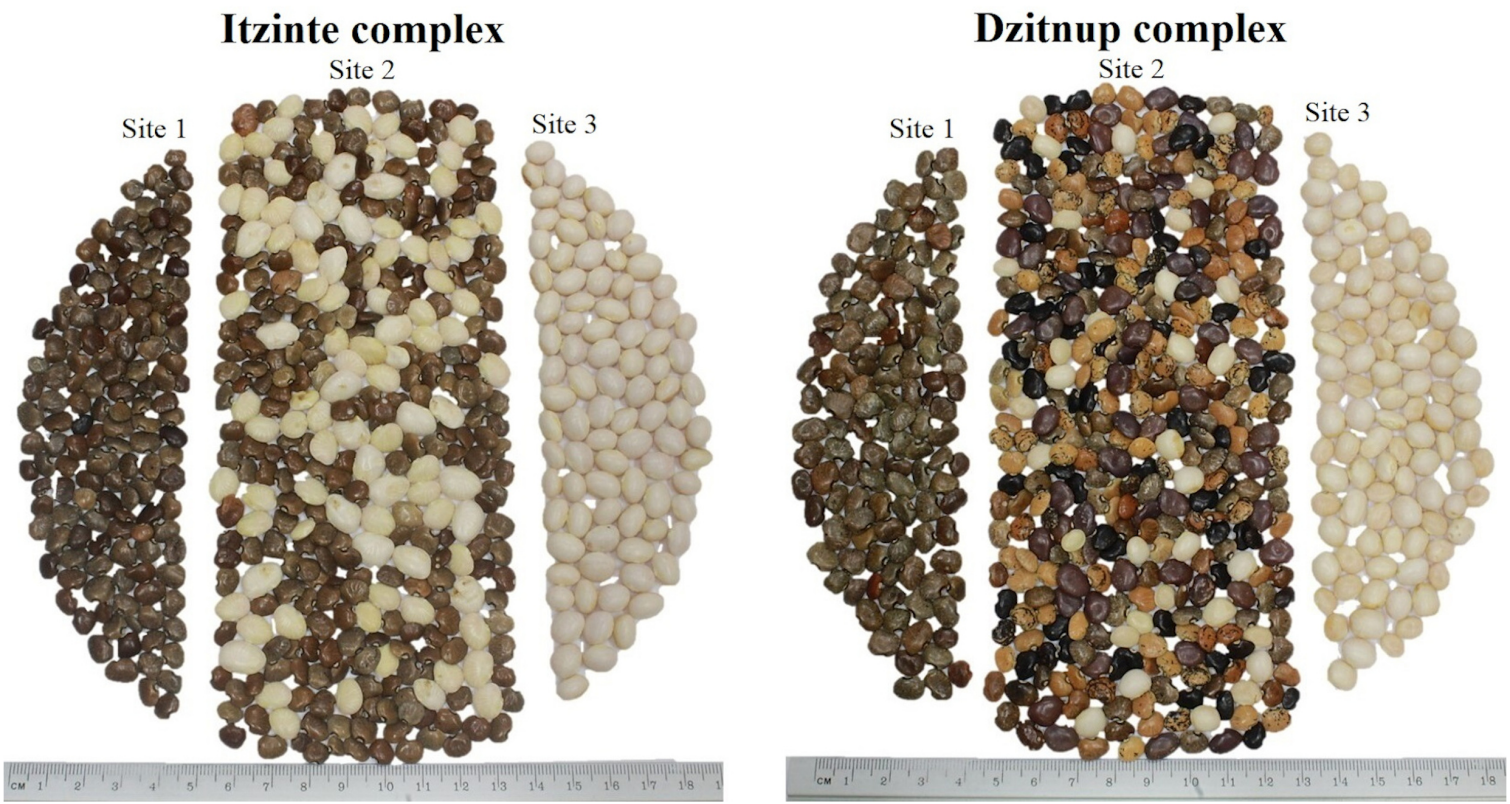
Collected seeds into the two wild-weedy-crop complexes studied. Photo taken by Pedro Ruiz-Gil.

Itzinté. Located in the abandoned Mayan ruins (N 20.018611 and W −89.724444; Fig. 1, Table S1), near de town of Bolonchén, located at the municipality of Hopelchén in the Campeche state. In this complex, wild and domesticated populations of Lima bean have coexisted for at least 20 years (Martínez-Castillo et al., 2004), but probably for longer, since this is an important cultivation area for farmers of Bolonchén, founded in 1957. Itzinté comprises an area of flat land interrupted only by mounds of the Mayan ruins and some hills, it has deep soils where the so-called continuous milpa is practiced (*i.e.,* plantation fields are used for many years continuously without a rest period). Within Itzinté, seeds were collected in three sites separated by a distance of 300–400 m: (a) site 1, the slope of a hill where only some wild plants were found; (b) site 2, a milpa where domesticated and wild plants were observed growing in close proximity (<1 m); and (c) site 3, a milpa with only one production cycle and whose seeds were brought from the town of Escárcega, located in the south of Campeche. A total of 27 seeds were sequenced from this complex, all from different plants: (a) three wild seeds from site 1, without morphological evidence of introgression; (b) 22 seeds from site 2, which spanned the entire range of morphological variation found and with possible introgression since they were collected from wild and domesticated plants growing near each other; and (c) two domesticated seeds from site 3, without morphological evidence of introgression (Fig. 2).

Dzitnup. Located near the town of Dzitnup, located in the municipality of Hecelchakán in Campeche state (Fig. 1, Table S1). Within this complex, seeds were collected in three sites separated by a distance of 1.5–2 km: (a) site 1, Xcalumkin archaeological zone (N 20.171806 and W −90.009722), where a wild population was found; (b) site 2, milpa located on the road from Dzitnup to Xcalumkin (N 20.163611 and W −90.05333), where domesticated and wild plants were growing intermingled or in close proximity (<1 m); the characteristics of the terrain (shallow soil with high stoniness) indicate that this is a milpa with 1–2 years of use; and (c) site 3, home garden at the exit of the town of Dzitnup (N 20.20011 and W −90.11611), heading for Xcalumkin. A total of 45 seeds from different plants were sequenced from this complex: (a) nine wild seeds collected at site 1, without morphological evidence of introgression; (b) 33 seeds collected at site 2, which spanned the entire range of morphological variation found and with possible introgression since they were collected from wild and domesticated plants growing near each other; and (c) three domesticated seeds collected at site 3, without morphological evidence of introgression (Fig. 2). In short, the SNP data set used for the analyses at the local scale considered 72 accessions (27 from Itzinté and 45 from Dzitnup).

(2) Regional scale

A total of 65 accessions in the broader Yucatan Peninsula were sequenced: 39 domesticated and 25 wild accessions; part of these accessions were collected in 2019 and others were obtained from the collection of Dr. Jaime Martínez-Castillo. In addition, we included data reported by Chacón-Sánchez & Martínez-Castillo (2017) comprising 13 domesticated and six wild accessions from this region, for a total of 83 accessions: 52 domesticated and 31 wild (Fig. 1, Table S2). Moreover, we incorporated a control group composed of samples from Chacón-Sánchez & Martínez-Castillo (2017) that did not include the Yucatan Peninsula: nine wild MI accessions (from Mexico), eight domesticated MI accessions (five from Mexico, two from Guatemala, and one from El Salvador), two wild MII accessions (one from Mexico and one from Guatemala), one domesticated MII accession from Costa Rica, and eight accessions from the Andean gene pool (five domesticated and three wild accessions from Ecuador and Peru) (Table S2). The purpose of incorporating the control group was to help in the assignment of individuals to one or several gene pools and in the prediction of chromosomal segments with possible introgression events. In short, the SNP data set used for the analyses at the regional scale considered 66 domesticated and 45 wild accessions.

### DNA extraction

Seedlings from the selected accessions were used to obtain DNA. Total genomic DNA was extracted from leaf tissue using a silica extraction protocol (Echevarría-Machado et al., 2005). DNA quality was verified by electrophoresis and visualization on 1% agarose gels, and DNA quantification was carried out in a Quantus ™ fluorometer. Genotyping-by-sequencing (GBS) library construction and 150 bp paired-end sequencing in an Illumina Hi-seq 2000 was performed at the Australian Genome Research Facility (Melbourne, Australia). Total genomic DNA extracted from each sample was digested with the *Ape* KI restriction enzyme which resulted in a population of fragments, most of them in the range of 200–300 bp. For library preparation, 100 ng of digested DNA and 3.6 ng of each adaptor were used for ligation. After ligation, 5 µl from each one of 96 libraries were mixed, amplified by PCR and sequenced in an Illumina sequencing lane. Average sequencing depth per accession ranged from 5X to 10X.

### SNP detection

The paired-end sequences obtained from the Illumina platform were de-multiplexed by their bar code using the Demultiplex option of the program NGSEP ver. 4.02 (graphical interface) (Duitama et al., 2014). Subsequently, data were cleaned using Trimmomatic v.0.36 (Bolger, Lohse & Usadel, 2014) to remove adapters and low-quality sequences (>Q30). Afterwards, reads were aligned to the Lima bean reference genome (available at: https://phytozome-next.jgi.doe.gov/info/Plunatus_V1) with the Bowtie2 tool and with default parameters in NGSEP (ver. 4.02); we then used the MultiSampleVariantsDetector option of NGSEP (ver. 4.02) to identify and genotype the genetic variants with the following parameters: 100 as the maximum number of alignments per start position, SNP heterozygosity rate of 0.0001 and minimum genotyping quality of Q40. The SNP matrix was filtered with the FilterVCF option of NGSEP (ver. 4.02) with the following options: MAF of 5%, missing data at a maximum of 20% (for loci and genotypes), minimum genotyping quality of Q40, maximum heterozygosity of 0.2 and removing SNPs from the repetitive regions of the genome. This filtering process resulted in 15,168 SNPs and 184 accessions. SNP density per chromosome varied from 15.2 SNPs/Mbp for chromosome PL10 to 41.8 SNPs/Mbp for chromosome PL03, with an average SNP density of 29 SNPs/Mbp. The VCF file was converted to other formats using TASSEL (Bradbury et al., 2007) and PGDSpider (Lischer & Excoffier, 2012).

Raw sequence data generated and used in this study have been deposited in the NBCI Sequence Read Archive (SRA) under the Bioproject accessions numbers PRJNA596114 and PRJNA823361. Details about the SRX number for each accession can be found in the Table S3. Also, data from both complexes analyzed and those from the Yucatan peninsula were included as Supplemental Information as a *.vcf file.

### Data analysis

#### Introgression at the local scale

The degree and direction of introgression within each wild-weedy-crop complex were evaluated by four methods, using all the 15,168 SNPs found:

(a) First, an individual assignment test based on global ancestry was applied, as implemented in STRUCTURE (Pritchard, Stephens & Donnelly, 2000). To assign individuals to either of two gene pools (wild and domesticated) we considered a percentage of own ancestry equal or greater than 90% to define genetically pure individuals. In this approach, individuals of admixed ancestry are indicative of potential introgression. The software was run with the admixture model and correlated allelic frequencies, with a burn-in period of 100,000 and 100,000 MCMC (Markov Chain Monte Carlo) iterations after burn-in, evaluating different K values (1–4) and running 5 independent simulations for each K value. To obtain the optimal K value, we used the method of Evanno, Regnaut & Goudet (2005) implemented in STRUCTURE-HARVESTER (Earl & VonHoldt, 2012). Then, we calculated the percentage of ancestry of each individual from both complexes by dividing the percentage of ancestry from the gene pool that does not correspond to the individual by the percentage of ancestry from the individual’s gene pool; to show these results we generated boxplot graphs with the R package ggplot2 (Wickham, 2016).

(b) Second, to compare the STRUCTURE results, a Principal Coordinate Analysis (PCoA) was performed using GenAlex (Peakall & Smouse, 2012).

(c) Third, a sliding-window analysis was applied using the IntrogressionAnalysis module of NGSEP 4.02 (Duitama et al., 2014), which allows predicting genomic regions with possible introgressed segments. The rationale behind this approach is that introgressed haplotypes should show higher genetic similarity to the putative source population than to the recipient population. This module divides the genome into non-overlapping windows of 50 SNPs to identify the most common haplotype within each of the populations described in the given populations file and then identify common haplotypes of one population introgressed in samples of a different population. For this analysis, the background population of each accession was assigned according to the STRUCTURE results, and the genomic regions with potential introgressions were called when the assigned population of a chromosomal segment (or haplotype) was different from the background population of the sample. Introgression events that spanned more than one 50 SNP window were retained, also single windows of 50 SNPs where the similarity index between the haplotype of the sample and the background population was below 15.

(d) Fourth, ABBA and BABA tests were carried out with the software Dsuite (Malinsky, Matschiner & Svardal, 2021). In this program we used the option Dtrios to calculate Patterson’s D and f4-ratio to detect and quantify introgression (admixture proportion), respectively, on a whole genome scale. We aimed to test whether wild (or domesticated) populations within each complex (Itzinté and Dzitnup) showed signals of introgression with sympatric domesticated (or wild) populations and in what proportion. For these tests, we evaluated topologies consistent with the Newick tree (((P1,P2)P3)O) where O is an outgroup assumed to be fixed for ancestral alleles (A alleles), P1 and P2 are sister groups, and P3 is a group used to test whether derived alleles (B alleles) present in P3 and shared with P1 (the BABA pattern) or P2 (the ABBA pattern) are the result of gene flow. Without gene flow, both patterns are equally likely and D equals zero, whereas with gene flow between P2 and P3 the ABBA pattern is more frequent, and D is positive. We tested eight gene flow models (4 models for each complex) to assess whether domesticated populations within the two complexes share more derived alleles with the wild populations within the same complex than with other wild populations in the Yucatan Peninsula (see Fig. S3). The significance of the statistics was assessed by jackknifing the data set into 20 blocks, and *p*-values at the 0.05 threshold were corrected by the Bonferroni method. After examining the results, we chose the models with the highest D values and f4-ratios to perform sliding window scans of introgression. For this, we used the Dinvestigate option in Dsuite to calculate the distance-based statistics df (distance fraction) (Pfeifer & Kapan, 2019) in windows of 10 informative SNPs moving by 1 SNP step. The df statistics combines the pairwise nucleotide distance (dxy) and Patterson’s D to detect and quantify introgression at fine genomic scales. With no gene flow, *df* = 0 (namely, the genetic distance between P1 and P3 equals the genetic distance between P2 and P3, d_13_ = d_23_), with gene flow between P2 and P3 df is positive (when d_13_ > d_23_), and with gene flow between P1 and P3 df is negative (when d_13_ < d_23_). Introgressed genomic regions (or blocks) were defined as those genomic windows within the top 10% of df outliers within each chromosome. Consecutive genomic windows complying with this definition were collapsed in the same introgressed block.

Genetic diversity was evaluated within each complex; individuals were grouped as wild or domesticated according to the percentage of ancestry obtained with STRUCTURE. Then, the number of polymorphic loci (P), the percentage of polymorphic loci (% P), the expected heterozygosity (*H*_E_) and the nucleotide diversity (*π*) were calculated using ARLEQUIN 3.5.22 (Excoffier & Lischer, 2010).

#### Structure, genetic diversity and gene flow at the regional scale

To determine the grouping pattern of the Lima bean accessions collected in the Yucatan Peninsula in relation to the control group, all the accessions considered in this study were characterized using three methods: (1) A Neighbor-Joining (N-J) analysis rooted with the Andean accessions and using DARwin V6 (Perrier & Jacquemound-Collet, 2006), (2) a Principal Coordinate Analysis (PCoA) using GenAlex (Peakall & Smouse, 2012), and (3) an individual assignment test with STRUCTURE (Pritchard, Stephens & Donnelly, 2000), following the previous specifications. Then, to determine the genetic structure of the Lima bean within the Yucatan Peninsula, another STRUCTURE and PCoA analyses were ran following the parameters indicated above, but without the control group; once the grouping pattern was determined, a molecular analysis of variance (AMOVA) was performed considering the different K values evaluated, using ARLEQUIN 3.5.22 (Excoffier & Lischer, 2010) and a hierarchical design that included the following sources of variation: between genetic groups, between individuals within genetic groups and within individuals.

Based on the genetic groups observed within the Yucatan Peninsula, the genetic diversity was evaluated using the next estimators: number of polymorphic loci (P), percentage of polymorphic loci (% P), expected heterozygosity (*H*_E_) and nucleotide diversity (*π*), calculated using ARLEQUIN 3.5.22 (Excoffier & Lischer, 2010).

Finally, the degree and direction of gene flow between the groups detected in the Yucatan Peninsula were determined using: (1) the STRUCTURE results obtained within the Yucatan Peninsula to evaluate recent gene flow; and (2) MIGRATE-N version 3.3 (Beerli, 2004) to evaluate historical gene flow. This program uses ratios of maximum likelihood to estimate migration rates based on the coalescent; to do this, we used the SNP analysis option with the following parameters: maximum likelihood inference, five short chains (10,000 steps) and three long chains (100,000 steps), for both chain lengths we used a 10,000 step burn-in and recorded the genealogy every 20 steps.

## Results

### Introgression at local scale

**Figure 3:**
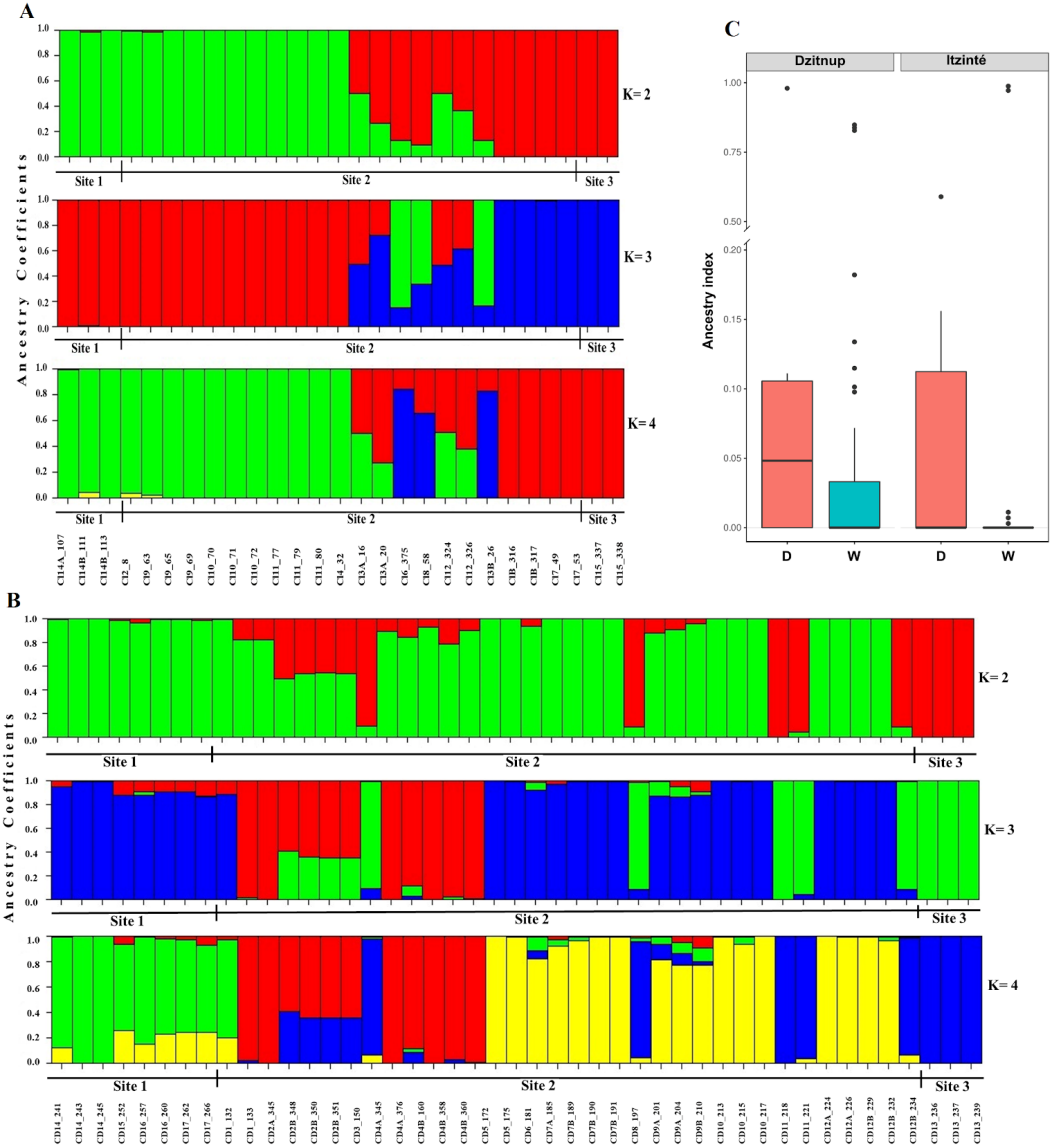
Estimated ancestry coefficients for two wild-weedy-crop complexes of Lima bean studied in the Yucatan Peninsula, using STRUCTURE and 15,168 SNP markers. (A) Itzinte; (B) Dzitnup; with optimal K = 2, green color represents the wild gene pool and red color represents the domesticated gene pool. (C) Ancestry index between domesticated (red) and wild (blue) accessions of Lima bean in Dzitnup and Itzinté complexes.

In Itzinté, optimal K was 2; STRUCTURE recognized genetically pure wild (green color) and domesticated (red color) individuals and identified seven individuals collected at site 2 that showed different grades of ancestry of both gene pools, which can be considered as admixed individuals (Fig. 3A). In Dzinup, K optimal was also 2; STRUCTURE recognized pure wild (green color) and domesticated (red color) individuals and identified 19 individuals collected at site 2 that showed ancestry of both gene pools, which can be considered as admixed individuals (Fig. 3B). Figure 3C showed that, in both complexes, for domesticated individuals the percentage of ancestry from the wild gene pool is, in most cases, low; on the contrary, in wild individuals, the percentage of ancestry from the domesticated gene pool is more variable and includes some individuals that show high values of ancestry from the domesticated gene pool. For Itzinté, the PCoA showed two compact groups of accessions (wild and domesticated), and some accessions with a scattered distribution that, according with STRUCTURE, correspond to admixed accessions; the first principal coordinate explained 54.13% of variation, followed by 7.27% for the second one, both coordinates explained 61.4% of the total variation (Fig. S1). Similarly, for Dzitnup, the PCoA showed two compact groups (wild and domesticated), with the rest of the accessions showing a scattered distribution (but minor compared with Itzinté) that, according with STRUCTURE, are also admixed accessions; the first principal coordinate explained 28.51% of variation, followed by 10.46% for the second one, both coordinates explained only 38.97% of the total variation (Fig. S2).

**Figure 4:**
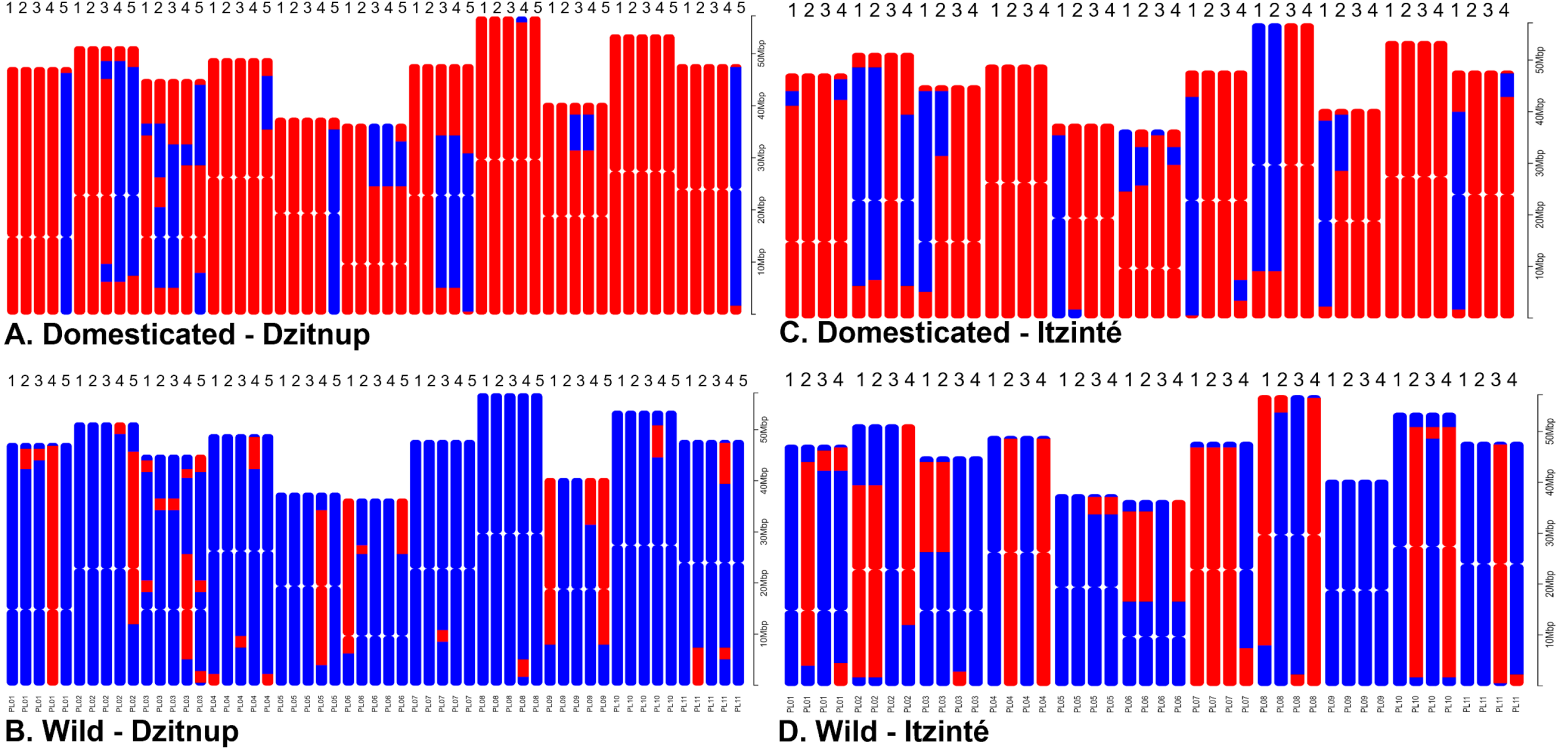
Distribution of introgression blocks per chromosome (PL01-PL11) detected by NGSEP. Dzitnup (A) and (B), Itzinté (C) and (D). Domesticated chromosomes are in red, wild chromosomes are in blue. Accessions shown in figure (A) 1. CD11_218, 2. CD11_221, 3. CD12B_234, 4. CD3_150, 5. CD8_197; in (B) 1. CD1_133, 2. CD12A_226, 3. CD15_252, 4. CD2B_350, 5. CD4B_358; in (C) 1. CI3B_26, 2. CI6_375, 3. CI7_49, 4. CI8_58; in (D) 1. CI12_324, 2. CI12_326, 3. CI13A_16, 4. CI13A_20.

For Itzinté, NGSEP identified 54 introgression events in four domesticated and seven wild individuals, all of them (except two) collected at site 2 (Figs. 4C and 4D; Table S4); these individuals corresponded with the seven admixed individuals observed with STRUCTURE. 33 of these events corresponded to introgressions from the domesticated to the wild gene pool. For Dzitnup, NGSEP identified 118 introgression events in 5 domesticated and 27 wild individuals, 26 of them collected at site 2 (Figs. 4A and 4B; Table S4); these individuals corresponded to 15 of the 19 admixed individuals observed with STRUCTURE. Of the 118 introgression events, 94 were in the domesticated to wild direction, and 24 events were in the opposite direction. It is worth mentioning that 11 wild individuals from Dzitnup share an introgression block from the domesticated gene pool at chromosome 3 of about 2 Mbp (from base 34,238,474 to base 36,221,738).

Although both STRUCTURE and NGSEP analyses indicated gene flow mainly from crop-to-wild in both complexes, these analyses also showed some evidence of gene flow in both directions. In order to delve into this, we conducted ABBA-BABA tests to assess whether the signals of introgression are stronger between wild and domesticated populations within the same complex than with other wild and domesticated populations of the Yucatan Peninsula. In effect, as it can be seen in Fig. S3 and Table S5, D values and f4-ratios are higher between domesticated and wild populations within the same complex (models m1, m3, m5 and m7) than with other wild/domesticated populations in the Peninsula of Yucatan (models m2, m4 and m6, except for model 8). The proportion of introgression, as measured by the f4-ratio, was higher in the older complex (for Itzinté was around 24%) than in the younger complex (for Dzitnup was around 15%).

To further investigate the introgression of domesticated alleles into wild populations (d-w), and vice versa (w-d), within both complexes at a fine genomic scale, we chose to investigate four models: m1, m3, m5 and m7. Fig. S4 and Table S6 show the distribution of D and df values in all eleven chromosomes in Itzinté and Dzitnup and the proportion of introgression in the direction wild to domesticated and vice versa. It can be observed that median df values for all four models were positive, indicating a higher contribution of domesticated/wild alleles in P2 (wild/domesticated populations within the same complex) than in P1 (wild MII or MI populations outside the complexes). For the older complex (Itzinté), median df values are very similar for models m1 and m3, indicating that the proportion of introgression is similar (df is around 12–13%) in both directions (d-w, w-d). In contrast, for the younger complex (Dzitnup) the proportion of introgression in the direction domesticated to wild (median *df* = 18%) is almost twice than in the opposite direction (median *df* = 10%). When comparing introgression proportions (median df) in both directions (d-w, w-d) at the chromosome level, df values are strikingly different in chromosome seven for Itzinté and in chromosome nine for Dzitnup, with df values larger in the direction domesticated to wild in both chromosomes (for details between the chromosomal regions found with evidence of introgression and its relation to domestication, see Table S6.1).

**Figure 5:**
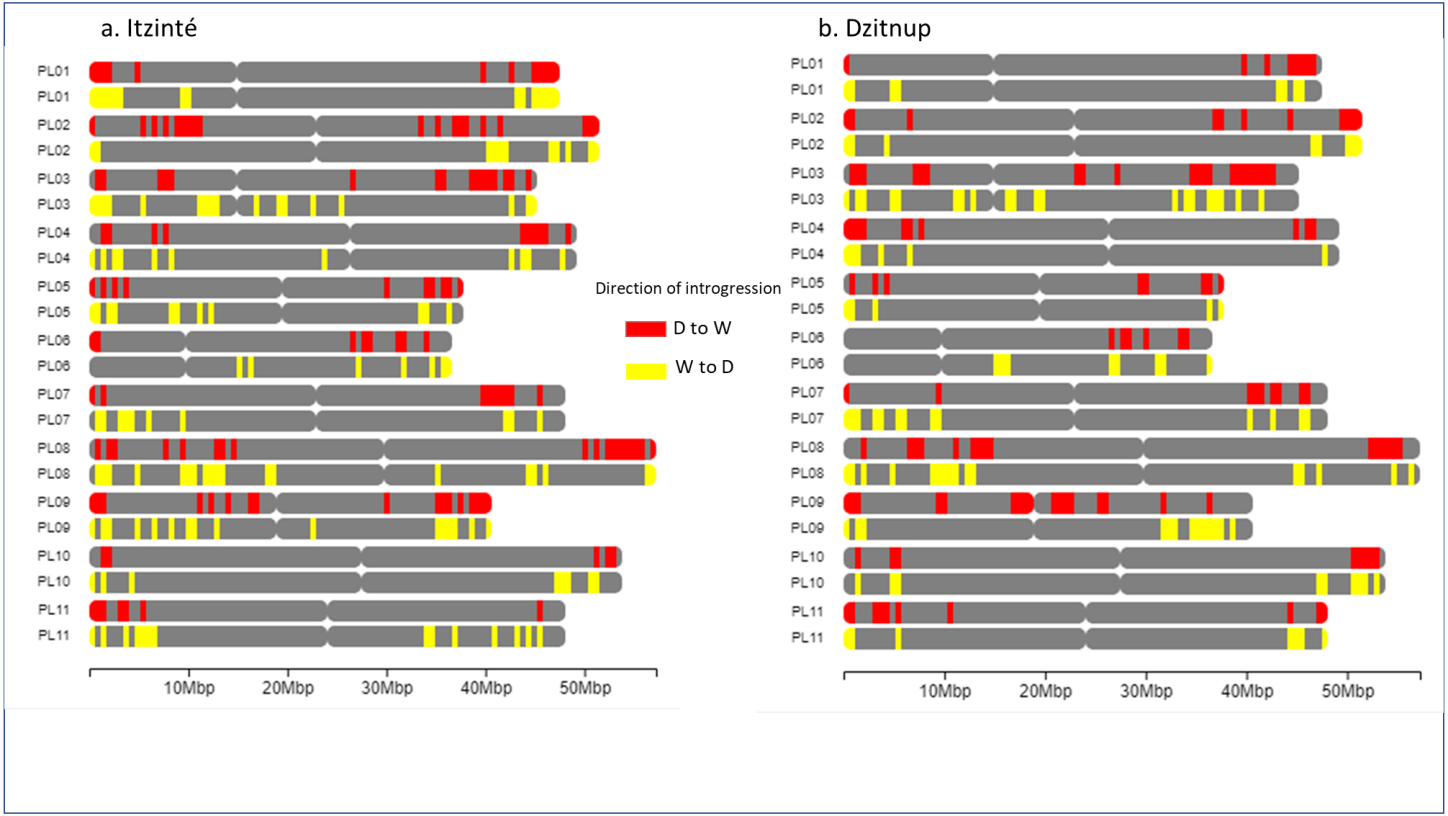
Distribution of introgression blocks along chromosomes. The figure shows the distribution of introgression blocks along chromosomes between wild and domesticated populations within (A) the Itzinté complex and (B) the Dzitnup complex. Red, introgression blocks in the direction domesticated to wild. Yellow, introgression blocks in the direction wild to domesticated. PL01-PL11, chromosomes.

Figure 5 shows the distribution of introgressed blocks in both complexes. It should be noted that introgression blocks in Fig. 5 are smaller than in Fig. 4 because Dsuite calculates introgression at the population level (not at the individual level as NGSEP does) and at a finer genomic scale in windows of 10 SNPs/1 SNP. In Itzinté (the older complex) the number of introgressed blocks was larger (189 blocks) than in Dzitnup (the younger complex) (129 blocks), and the size of the genome occupied by all the introgressed blocks was larger in Itzinté than in Dzitnup (195 Mb *versus* 158 Mb) (Tables S7–S8). In contrast, the average size of the individual blocks was larger in Dzitnup (1.2 Mb/block) than in Itzinté (1.0 Mb/block). All this indicates that in the older complex, the introgressed blocks are more in number but smaller in length compared to the younger complex. Also, for Dzitnup the size of the genome occupied by introgressed blocks was larger in the direction d-w (88 Mb) than in the w-d direction (70 Mb), suggesting gene flow may be predominant from landraces to wild populations in this complex. The opposite pattern occurred in Itzinté (76 Mb and 119 Mb, respectively), suggesting gene flow may be predominant from wild to landrace populations.

In relation to the genetic diversity found in both complexes, *H*_E_ values were slightly higher in the domesticated accessions (Dzitnup = 0.36; Itzinté = 0.34) than in the wild ones (Dzitnup = 0.28, Itzinté = 0.31); but the other estimators were greater in the wild accessions than in the domesticated ones (Table 1), although the statistical significance for these analyses was not evaluated.

**Table 1:** Genetic diversity of Lima bean (*Phaseolus lunatus* L.) in the Yucatan Peninsula, Mexico, at two scales and using 15,168 SNP markers.

**Scales**	***N***	***P***	***%P***	***H*_**E**_**	*π*
**Local**					
*Dzitnup complex*					
Wild	32	4124	27.2	0.2791 ± 0.3976	0.0819 ± 0.0392
Domesticated	8	1822	12	0.3609 ± 0.3606	0.0471 ± 0.0238
*Itzinte complex*					
Wild	14	2081	13.7	0.3087 ± 0.3961	0.0449 ± 0.0220
Domesticated	9	1763	11.6	0.3363 ± 0.3562	0.0420 ± 0.0210
**Regional**					
Wild pure group	15	1586	10.4	0.3647 ± 0.1404	0.0401 ± 0.0195
Wild admixed group	17	2076	13.6	0.2597 ± 0.1538	0.0666 ± 0.0324
Domesticated group	51	727	4.7	0.2344 ± 0.1542	0.0124 ± 0.0059

**Notes.**

N sample size P polymorphic loci number %*P* polymorphic loci percentage *H*_E_ expected heterozygosity π nucleotide diversity

### Structure, genetic diversity and wild-crop gene flow in Lima bean at regional scale

**Figure 6:**
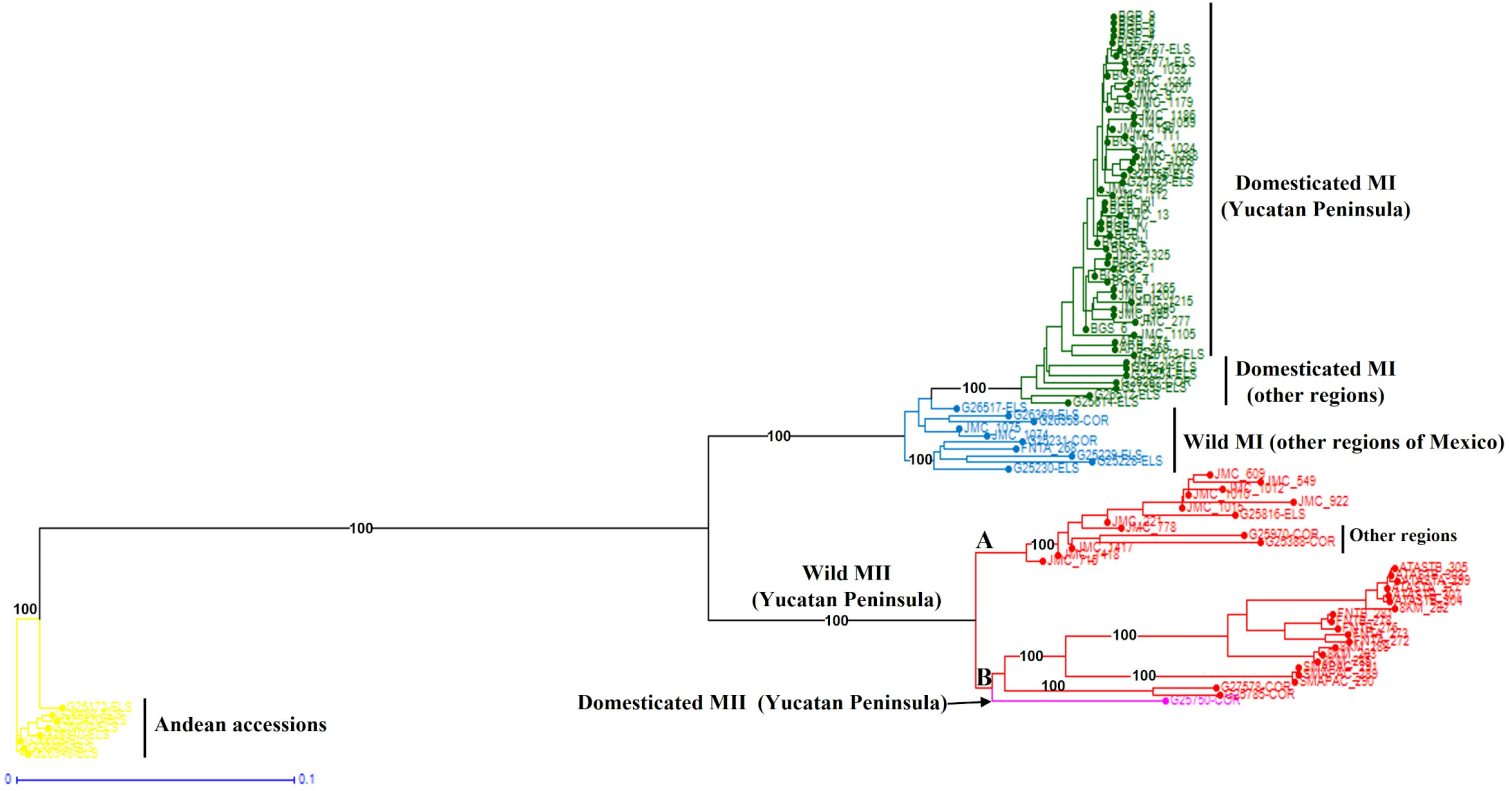
Neighbor-Joining analysis of Lima bean accessions from the Yucatan Peninsula, including a control group, using 15,168 SNP markers.

When the Lima bean accessions of the Yucatan Peninsula were analyzed together with the control group accessions, the Neighbor-Joining (N-J) analysis (Fig. 6) clearly separated all the accessions in two groups, according to the main gene pools of Lima bean: MI and MII. Into the MI group, two subgroups were observed: the green one integrated for all domesticated accessions from the Yucatan Peninsula and those collected in other regions of Mexico and central America, and the blue subgroup integrated for all wild accessions collected out of the Yucatan Peninsula. Into the MII group (indicated in red), we also observed two subgroups: one composed of 12 accessions from the Yucatan Peninsula plus one accession from central Mexico and another from Costa Rica; and another composed of 19 wild accessions collected in Tabasco and Campeche, one collected in Guatemala, plus a domesticated accession collected in Campeche. The Principal Coordinate Analysis (PCoA) (Fig. S5) generated a grouping pattern similar to that obtained with the N-J analysis. The first principal coordinate explained 45.69% of variation, followed by 17.02% for the second one; together, both coordinates explained 62.71% of the total variation. The optimal K obtained was 2; STRUCTURE analysis with *K* = 2 (Fig. S6A) grouped the wild MII accessions collected in the Yucatan Peninsula with the Andean accessions (green group), while all wild and domesticated accessions of the MI group were grouped together (red group). STRUCTURE with *K* = 3 (Fig. S6B) grouped the accessions according to the three large gene pools of Lima bean: MII (red group), MI (blue group) and Andean (green group). Finally, when STRUCTURE was run with *K* = 4 (Fig. S6C), the clustering pattern generated resulted very similar to the obtained with the N-J and the PCoA analyses.

**Figure 7:**
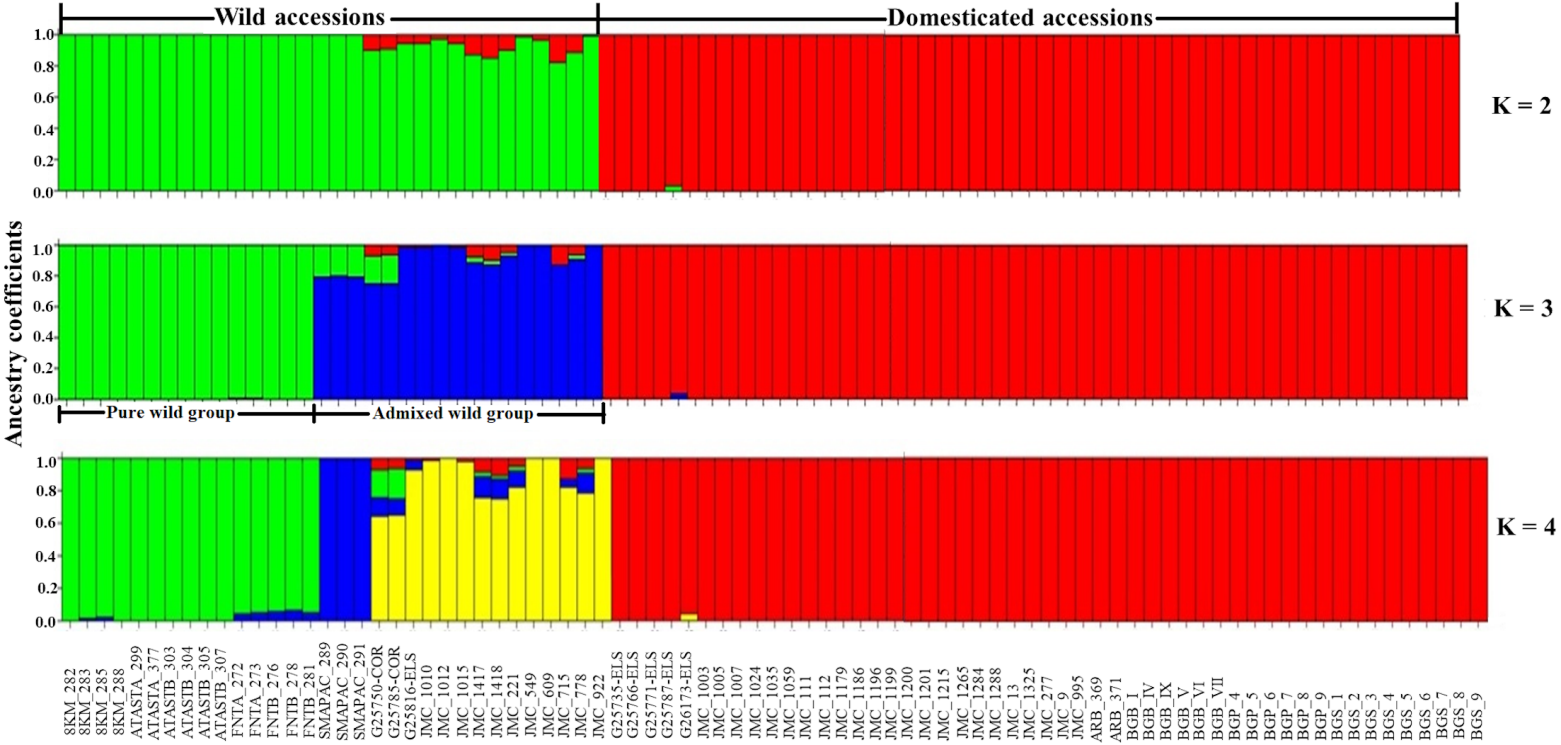
Estimated ancestry coefficients for wild and domesticated Lima bean accessions from the Yucatan Peninsula, using STRUCTURE and 15,168 SNP markers. With optimal K = 3, green represents the Pure wild group, blue represents Admixed wild group and red represents the domesticated group.

Considering only the Lima bean accessions of the Yucatan Peninsula, the optimal K obtained was 3; STRUCTURE separated the domesticated accessions (all integrated in one group) from the wild ones (integrated in two groups) (Fig. 7): a pure wild group was integrated by 15 wild accessions collected in Tabasco and western Campeche, and an admixed wild group was integrated by 17 wild accessions collected in Yucatan, Quintana Roo and central-eastern Campeche. PCoA showed the existence of three groups (Fig. S7), supporting the STRUCTURE results; the first principal coordinate explained 61.51% of variation, followed by 9.97% for the second one, both coordinates explained 71.48% of the total variation. Likewise, the AMOVA supported the existence of three groups, showing that the strongest genetic differentiation is found between groups (81.74%) (Table S8).

The pure wild group from the Yucatan Peninsula showed a greater value of *H*_E_ (0.36) than the admixed wild group (*H*_E_ = 0.26), and the latter showed a slightly higher value than the domesticated group (*H*_E_ = 0.23); the admixed wild group showed higher values for the other estimators (P% and *π*), and the domesticated group showed the lowest values for all estimators (Table 1). Statistical differences were not analyzed.

In relation to the recent wild-crop gene flow in the Lima bean of the Yucatan Peninsula, STRUCTURE (*K* = 3) separated the wild accessions from the domesticated ones, with most accessions showing high ancestry coefficients (>90%). With *K* = 3, a group of wild accessions considered as genetically pure were collected in Tabasco and western Campeche where Lima bean is not planted and clustered apart from another group of wild accessions that showed some ancestry from the domesticated gene pool, and that were collected in areas of traditional agriculture of Yucatan, Quintana Roo and central-eastern Campeche, where wild and domesticated Lima bean populations grow in sympatry. Moreover, only three domesticated accessions showed some degree of ancestry from the wild gene pool, although it was low (Fig. 7).

Taking in account the existence of three genetic groups into the Yucatan peninsula, MIGRATE showed a higher historical mutation scaled immigration rate (M = m/µ) from domesticated group (157.17) to admixed wild group (77. 07), slightly higher from the pure wild group (130.61) to admixed wild group (123.14), and higher from the domesticated group (114.07) to the pure wild group (55.93).

## Discussion

### Introgression and gene flow in Lima bean of the Yucatan Peninsula

STRUCTURE and NGSEP analyses showed predominantly a crop-to-wild introgression at the local scale, but also some evidence of a bidirectional gene flow in both studied complexes; ABBA-BABA tests showed bidirectional gene flow in both complexes, but predominantly in the direction wild-to-crop in Itzinté and in the opposite direction in Dzitnup. These tests also showed a higher introgression proportion in Itzinté than in Dzitnup.

Itzinté is the oldest of the two complexes, it has existed for at least 20 years (Martínez-Castillo et al., 2004), suggesting that the wild-crop gene flow could have been occurring continuously during this period. There, the practice of continuous milpa has generated an environment in permanent disturbance that has favored the presence of wild populations due to two characteristics of these ones: (1) a metapopulation dynamic (Barrantes et al., 2008), which has been identified as one of the factors that affect the dynamics of wild-crop introgression (Meirmans, Bousquet & Isabel, 2008; Ellstrand et al., 2013); and (2) a seed bank with an up to three years viability (Degreef et al., 2002). In this regard, some models predict that the dynamics of the seed bank and the establishment of seedlings under competition represent the strongest determinants of the persistence of weedy populations that occupy disturbed areas (Linder & Schmitt, 1994; Linder & Schmitt, 1995). In Itzinté, the fate of the resulting introgressed seeds can follow two possible scenarios: (1) if human selection is exerted, both directly on the plant material or indirectly by significant modification of the environment, the complex will progressively include only domesticated-type individuals; if farmers benefit from the generated variants, the presence of this type of complexes could be indicative of an on-going domestication process (Beebe et al., 1997); and (2) plants within the complex could evolve into wild forms under natural selection; it is known that selection within hybrid zones can act in two ways: (a) endogenous selection (not dependent on the environment), which assumes that hybrid generations may have inherent and consistent fitness advantages or disadvantages in relation to their parents and each other, and (b) exogenous selection (dependent on the environment), which considers that selection operating on hybrids may differ according to environmental conditions, implying the existence of genotype-environment interactions (Campbell & Waser, 2007; Kimball, Campbell & Lessin, 2008).

In Dzitnup, the wild-crop contact site was located in a slash-and-burn milpa. This type of milpa is commonly used only for 1–3 years and then left for a rest period (fallow) that can last up to 15 years (Hernández-Xolocotzi, 1959). It means that this complex will not remain for long, perhaps 2–3 years while the milpa is in use and one more year after production ends, allowing not harvested seeds to fall, germinate, reach the reproductive stage and breed with wild plants; it is the most common scenario for wild-crop introgression events in Lima bean of the Yucatan Peninsula. Characteristics of this complex suggests two possible scenarios for the resulting introgressed seeds: (1) if the seeds are harvested, they will be subject to human selection; current knowledge indicates that the genetic basis of domestication traits involve generally few genomic regions and therefore can be purged quite rapidly (Papa et al., 2005; Papa et al., 2007); and (2) if the introgressed seeds remain in the field, natural selection will act on the resulting hybrids and their maintenance will depend on their fitness (Mercer et al., 2014).

In both complexes, the identified introgressed individuals were mostly collected at the sites where wild and domesticated plants grew very close to each other (sites # 2). In hybrid zones, the relative frequencies of hybrid *versus* non-hybrid genotypes are expected to vary according to some characteristics, including distance between hybridizing individuals, relative size of each population, overlap in the flowering period, and pollen and seed dispersal (Nagy, 1997; Ellstrand, 2003; Jenczewski, Ronfort & Chévre, 2003). These characteristics were not evaluated in our study, but we observed that the wild and domesticated individuals in Dzitnup grew intermingled, while in Itzinté both types of individuals grew, mainly, in adjacent places. In relation to the flowering period of wild and domesticated populations of Lima bean, they overlap throughout the Yucatan Peninsula (Martínez-Castillo et al., 2004) and it is estimated that pollen and seed dispersal does not exceed 6 m, with some exceptions (Hardy et al., 1997; Baudoin et al., 1998); furthermore, both complexes were located in the region known as Los Chenes, Campeche, separated by approximately 40 km, and under very similar ecological-environmental conditions.

The asymmetry in the wild-crop introgression found at local scale (mainly crop-to-wild in Dzitnup, where more wild accessions were analyzed than domesticated ones, compared with Itzinté), is expected when the relative size of wild or domesticated populations is different, or when populations are fragmented (Ellstrand, 1992; Ellstrand, Prentice & Hancock, 1999); unfortunately, we did not evaluate these factors. Dzul-Tejero, Coello-Coello & Martínez-Castillo (2014), evaluated introgression in three Lima bean complexes in the Yucatan Peninsula using 11 SSR loci and ancestry coefficients obtained with STRUCTURE; these authors reported significant differences in the degree of introgression among the three complexes studied, the complex with the least introgression was a milpa where the farmer recognized and eliminated introgressed seeds, while the complex with the highest introgression was a milpa where the farmer recognized and positively selected the introgressed seeds; but did not find an asymmetry within any of the complexes. However, in this study we used a wider genomic sampling (15,168 SNPs) and the detection of introgression events at a finer level (chromosome); furthermore, the two studies were conducted in different areas of the Yucatan Peninsula and did not involve the same wild populations or the same landraces, so different factors (*e.g.*, agricultural, genetic or environmental management) may explain the differences observed in both studies.

At the regional scale, gene flow showed a crop-to-wild asymmetry, according to STRUCTURE; however, this asymmetry was detected only in wild accessions that were collected in areas of traditional agriculture where wild and domesticated populations coexist, since the wild accessions collected in Tabasco and western Campeche (where Lima bean is not planted) did not showed genetic infiltration of the domesticated gene pool. This crop-to-wild asymmetry was also supported with the results from MIGRATE, which showed more historical gene flow from the domesticated gene pool to the wild one; the admixed wild group appears to be acting as a genetic bridge between the domesticated populations and the pure wild populations. Martínez-Castillo et al. (2007), using nine loci of SSRs, also reported an asymmetric historical gene flow in Lima bean from the Yucatan Peninsula, three times greater from the domesticated towards the wild gene pool. The crop-to-wild asymmetry observed in Lima bean has also been reported in common bean (*Phaseolus vulgaris* L.) from other regions of Mexico and the Americas (Beebe et al., 1997; Papa & Gepts, 2003; Papa et al., 2005; Zizumbo-Villarreal et al., 2005; Hoc et al., 2006).

Crop-to-wild gene flow and introgression asymmetry, as observed at the regional scale (and into Dzitnup), may depend on the fitness of several hybrid generations in relation to their wild counterpart (Ellstrand et al., 2013), which can also be strongly influenced by the environment (Mercer, Wyse & Shaw, 2006). In species such as Lima bean, different aspects related to agricultural management could also help explain the asymmetry: (1) In cultivated environments, farmers who grow common bean easily recognize and select against F1 hybrids, since its seeds generally have an intermediate size compared to the parentals, and a different color compared to the seeds of the domesticated maternal plant; however, in wild environments, the domesticated traits tend to be hidden by the wild alleles and natural selection will only operate in later generations (Papa & Gepts, 2003; Papa et al., 2005). In Lima bean, the recognition of hybrids is also based on the flavor of the harvested seeds. Wild seeds contain high concentrations of linamarin, a cyanogenic glycoside that makes them inedible (Maquet, 1991) and whose presence allows Mayan farmers to detect unpleasant flavors in the introgressed seeds of their landraces (Martínez-Castillo et al., 2007). (2) The intensification of traditional agriculture in the Yucatan Peninsula has generated an increase in the use of herbicides, a decrease in the rest period, the presence of maize monocultures and the use of agricultural machinery, among other changes (Martínez-Castillo et al., 2004). These changes can generate a reduction in density of wild populations present in domesticated areas, thereby favoring the existence of a higher pollen production of domesticated plants and an increased gene flow to wild plants that remain (Papa & Gepts, 2003; Papa et al., 2005). In the Yucatan Peninsula, some wild individuals have been observed growing near or among hundreds of domesticated Lima bean plants (Martínez-Castillo et al., 2004). (3) In some cities of the Yucatan Peninsula, landraces of Lima bean with white seeds and sweet flavors are preferred, which favors their cultivation; this has led producers in southern Yucatan to eliminate wild populations to avoid introgression with their landraces and thus obtain better sales (Martínez-Castillo et al., 2004); this could help explain the lower degree of genetic infiltration in the domesticated populations of southern Yucatan (Martínez-Castillo et al., 2007).

Several studies have reported the negative aspects of a crop-to-wild asymmetry in relation to the conservation of plant genetic resources, among these, the evolution of new weeds or invasive species (Schierenbeck & Ellstrand, 2009) contributing to the risk of local extinction (Ellstrand & Elam, 1993; Levin, Francis-Ortega & Jansen, 1996), as well as to the risk associated to transgene escape (Gepts & Papa, 2003; Jenczewski, Ronfort & Chévre, 2003; Pilson & Prendeville, 2004; Andow & Zwahlen, 2006). Considering the characteristics of the Mayan milpa, the risk of local extinction of wild Lima bean populations can be considered low, since the dynamics of the milpa allows their existence, which is also favored by their metapopulation behavior. Martínez-Castillo et al. (2006) reported the existence of a positive correlation between agricultural intensification and increase in the genetic diversity of the Lima bean populations, suggesting that these populations are favored by the intensification of disturbance in situations involving at least three years of fallow. Even in complexes like Itzinté (where there is a permanent wild-crop contact), where wild populations could show a greater risk of local extinction due to swamping (*i.e.,* which occurs when a small population loses its genetic integrity and assimilates into a larger population, as a result of repeated episodes of hybridization and introgression; (Ellstrand, Prentice & Hancock, 1999), our results showed high levels of genetic diversity in the wild population, higher than those showed at the regional level. Regarding the negative effects of transgenic crops on wild relatives, it is known that the existence of gene flow and a crop-to-wild asymmetry, as we showed happens in the Lima bean from de Yucatan Peninsula, is necessary for transgene escape (Gepts & Papa, 2003). Currently there are no transgenic varieties of Lima bean, but our study and those of Martínez-Castillo et al. (2007) and Dzul-Tejero, Coello-Coello & Martínez-Castillo (2014) warn of the risks of their possible release in the Yucatan Peninsula.

Contrary, wild-crop introgression may have positive effects on the evolution of domesticated species (Harlan, 1965; Jarvis & Hodgkin, 1999), and wild-weedy-crop complexes are of great importance in the generation of genetic variability in landraces (Pernès & Lourd, 1984; Harlan, 1992; Beebe et al., 1997). Introgression represents one of the main sources of variation for genetic improvement of crops, including that carried out by farmers in traditional agroecosystems (Hajjar & Hodgkin, 2007; Worthington et al., 2012). For Lima bean from the Yucatan Peninsula, high levels of genetic diversity have been reported in individuals grown from milpas with high levels of introgression, compared to milpas with low introgression, as well as the selection and consumption of weedy variants by Mayan farmers (Dzul-Tejero, Coello-Coello & Martínez-Castillo, 2014). This evidence could help explain why the Yucatan Peninsula is the region with the highest number of landraces in Mexico and a center of genetic diversity for this species in Mesoamerica (Ballesteros, 1999; Martínez-Castillo et al., 2004).

### Genetic structure and diversity of Lima bean in the Yucatan Peninsula

Using control groups for some analyses helped in the assignment of the individuals collected in the Yucatan Peninsula to the main gene pools of Lima bean (MI or MII); however, these analyses masked the existing genetic structure within this region (in particular, in the wild gene pool where two groups were found). Although we detected a group of admixed wild individuals, Lima bean in the Yucatan Peninsula showed a strong genetic structure based on the existence of wild MII and domesticated MI gene pools. This is an interesting result considering that gene flow and introgression processes between wild and domesticated Lima bean may have been occurring for many centuries in this region as revealed by archaeological evidence (Kaplan & Kaplan, 1988). Even though the self-pollinated tendency of Lima bean limits wild-crop gene flow and could thus explain the observed genetic structure, our study and that of Dzul-Tejero, Coello-Coello & Martínez-Castillo (2014) clearly show the existence of introgression events at a local scale, suggesting that selection is playing an important role in maintaining the genetic identity of wild and domesticated populations that grow in sympatry, as has been reported for common bean (Papa & Gepts, 2003).

In the Yucatan Peninsula we found higher *H*_E_ values in the wild gene pool (especially in the Pure wild group) than in the domesticated one. In contrast, although *π* values were higher for the wild gene pool compared with the domesticated one, for this estimator the Admixed wild group showed a higher nucleotide diversity than the Pure wild group, probably due to the higher percentage of polymorphic sites in the Admixed wild group. Although *H*_E_ is a good estimator because it is directly related to genetic diversity and is not influenced by differences in sample size (such as P%), the differences found in *H*_E_ values should be taken with caution considering that the statistical significance of this estimator was not evaluated among groups. Differences in genetic diversity between crops and their wild relatives can be explained by a founder effect due to domestication (Ladizinsky, 1985) (and subsequent introduction of the crop into the Peninsula), which has already been reported for this species using SNPs (Chacón-Sánchez & Martínez-Castillo, 2017), ribosomal DNA (Serrano-Serrano et al., 2012) and chloroplast DNA (Andueza-Noh et al., 2013). Chacón-Sánchez & Martínez-Castillo (2017), using 4,593 SNP markers and individuals from the entire Lima bean distribution area in the Americas, reported *H*_E_ values that are lower (wild MII = 0.138, domesticated MI = 0.079) than those found by us; although both studies considered different geographical scales, our results suggested that the Yucatan Peninsula is an important area of genetic diversity for Lima bean. Relatively high values of genetic diversity for wild and domesticated populations of Lima bean (considering its endogamous nature) from the Yucatan Peninsula have also been reported using SSR (Martínez-Castillo et al., 2006) and ISSRs (Martínez-Castillo, Colunga-GarcíaMarín & Zizumbo-Villarrreal, 2008; Camacho-Pérez et al., 2017) markers. Even though our study showed a crop-to-wild asymmetry in Lima bean at regional scale, which was also reported by Martínez-Castillo et al. (2007), we did not find evidence of a negative impact on the genetic diversity of the wild gene pool, as expected in crop-to-wild gene flow (Ellstrand, Prentice & Hancock, 1999).

## Conclusions

This study provides important elements to understand the evolutionary dynamics of Lima bean in the Yucatan Peninsula, its Mesoamerican diversity center, using a broad genomic sampling of the wild and domesticated gene pools of the species. Our results suggest that gene flow and introgression can be playing an important role at the local scale, but its consequences on the structure and genetic diversity of Lima bean are not clearly reflected at the regional scale, where diversity patterns between wild and domesticated populations could be reflecting historical events instead. Even when evidence of a bidirectional gene flow was found at the local scale, as well as an asymmetry in crop-to-wild introgression in Dzitnup, these processes do not seem to be affecting the genetic structure and diversity of the species in the longer term. We observed a marked genetic differentiation between the wild MII and domesticated MI gene pools at the regional scale, with the former showing higher genetic diversity, which is common in annual crops that have undergone a strong founder effect due to domestication. This study also provides valuable information for the conservation of wild Lima bean: wild populations that come into contact with domesticated populations for a short period of time (such as Dzitnup), do not present a high risk of local extinction, given that once milpas are abandoned, the generated hybrids will likely be eliminated by natural selection in a few generations. Even in Itzinté, where wild population maintain permanent contact with a domesticated population, does not show risk of local extinction due to swamping. Results from this study can also be useful for the genetic improvement of Lima bean in traditional agroecosystems. The existence of wild-weedy-crop complexes opens the possibility for farmers to benefit from the weedy variants; the presence of this type of complexes could be indicating an on-going domestication process.

## Supplemental Information

10.7717/peerj.13690/supp-1 Figure S1 Analysis of Principal Coordinates (PCoA) of Lima bean from the Itzinté complex, using 15,168 SNP markers The colors assigned to the observed groups correspond to those used in the STRUCTURE analysis with *K* = 2 (Fig. 3-A).

10.7717/peerj.13690/supp-2 Figure S2 Analysis of Principal Coordinates (PCoA) of Lima bean from the Dzitnup complex, using 15,168 SNP markers The colors assigned to the observed groups correspond to those used in the STRUCTURE analysis with *K* = 2 (Fig. 3-B).

10.7717/peerj.13690/supp-3 Figure S3 Models used for the ABBA-BABA tests In these models, wild MII populations from Mexico (outside the Yucatan Peninsula) were assigned as the sister group (P1) of wild populations within the complexes or within the Yucatan Peninsula assigned as P2, and wild MI populations from Mexico were assigned as the sister group (P1) of domesticated populations within the complexes or within the Yucatan Peninsula assigned as P2. In each model P3, the tested group, was appropriately chosen to test gene flow between P3 and P2. In all these models, the Andean wild gene pool (AI) was fixed as outgroup.

10.7717/peerj.13690/supp-4 Figure S4 Distribution of df values per chromosome The figure shows the proportion of introgression (df) per chromosome in the Itzinté (a) and Dzitnup (b) complexes in the direction domesticated to wild (d-w) (red bars) and wild to domesticated (w-d) (yellow bars). Gray dashed lines indicate df = 0, red and yellow dashed lines indicate median df values observed in the direction d-w and w-d, respectively.

10.7717/peerj.13690/supp-5 Figure S5 Analysis of Principal Coordinates (PCoA) of Lima bean from the Yucatan Peninsula and the control group, using 15,168 SNP markers The colors assigned to the observed groups correspond to those used in the Neighbor-Joining analysis (Fig. 6).

10.7717/peerj.13690/supp-6 Figure S6 Estimated ancestry coefficients for wild and domesticated Lima bean accessions from the Yucatan Peninsula and considering the control group, using STRUCTURE and 15,168 SNP markers The colors correspond to the observed groups using different K values: (A) K = 2; (B) K = 3; (C) K = 4. Colors with K = 4 match to those used in the Neighbor-Joining analysis (Fig. 6) and in the PCoA (Fig. S5).

10.7717/peerj.13690/supp-7 Figure S7 Analysis of Principal Coordinates (PCoA) of Lima bean using only accessions collected in the Yucatan Peninsula and 15,168 SNP markers The colors assigned to the observed groups correspond to those used in the STRUCTURE analysis with K = 3 (Fig. 7).

10.7717/peerj.13690/supp-8 Table S1 Data of accessions collected within Dzitnup and Itzinté complexes

10.7717/peerj.13690/supp-9 Table S2 Data of accessions used at the regional level (Table S2), Models used for the ABBA-BABA tests (Table S5). D and df values evaluating introgression between wild and domesticated populations within the complexes Itzinté and Dzitnup (Table S6)

10.7717/peerj.13690/supp-10 Table S3 Genbank Accession Numbers: GBS Data

10.7717/peerj.13690/supp-11 Table S4 NGSEP results with the two complexes studied

10.7717/peerj.13690/supp-12 Table S6 S6.1, Chromosomal regions with evidence of introgression and location of domestication QTL and S8, Number of introgression bolcks unique and shared among the Dzitnup and Itzinté complexes

10.7717/peerj.13690/supp-13 Supplemental Information 13 SNP data of Dzitnup complex

10.7717/peerj.13690/supp-14 Supplemental Information 14 SNP data of Itzinté complex

10.7717/peerj.13690/supp-15 Supplemental Information 15 SNP data of Yucatan peninsula

## Additional Information and Declarations

### Field Study Permissions

The following information was supplied relating to field study approvals (*i.e.*, approving body and any reference numbers): The seed collection was carried out with the approval of the CICY Herbarium, wich has permission from Secretaria de Medio Ambiente y Recursos Naturales (SEMARNAT)-Mexico (SGPA/DGVS/008421/18).

### Data Availability

The following information was supplied regarding data availability: The raw sequence data are available in the NBCI Sequence Read Archive (SRA): PRJNA596114 and PRJNA823361. Further information about the SRX number for each accession is available in Table S3.
