# Supplementary figures and images for: Consequences of introgression and gene flow on the genetic structure and diversity of Lima bean (Phaseolus lunatus L.) in its Mesoamerican diversity area
Source: PeerJ. 2022 Jul 5;10:e13690. doi: 10.7717/peerj.13690 (PMC9266586; doi:10.7717/peerj.13690)

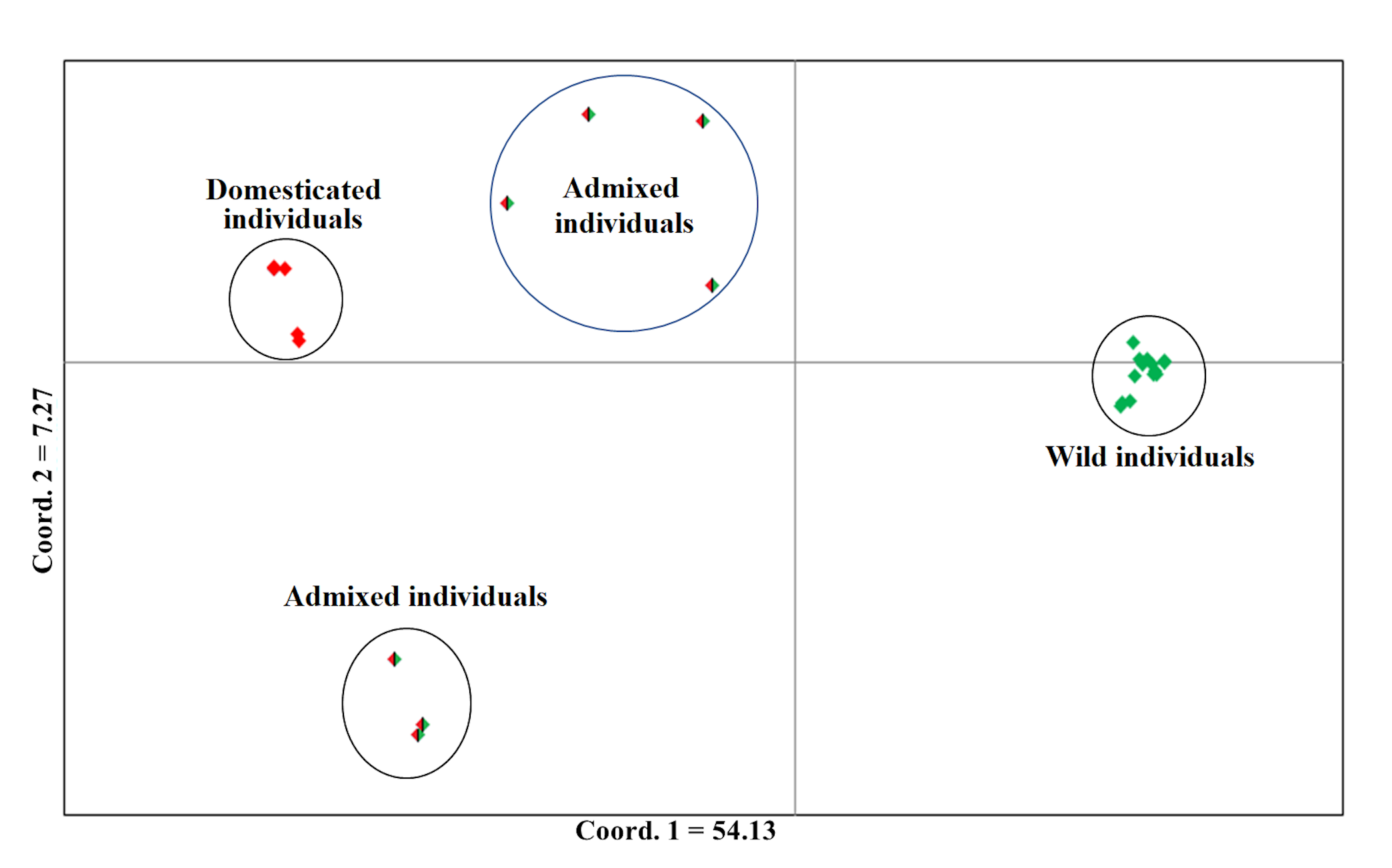

Supplement: Figure S1 — The colors assigned to the observed groups correspond to those used in the STRUCTURE analysis with K = 2 (Fig. 3-A). [file peerj-10-13690-s001.png]

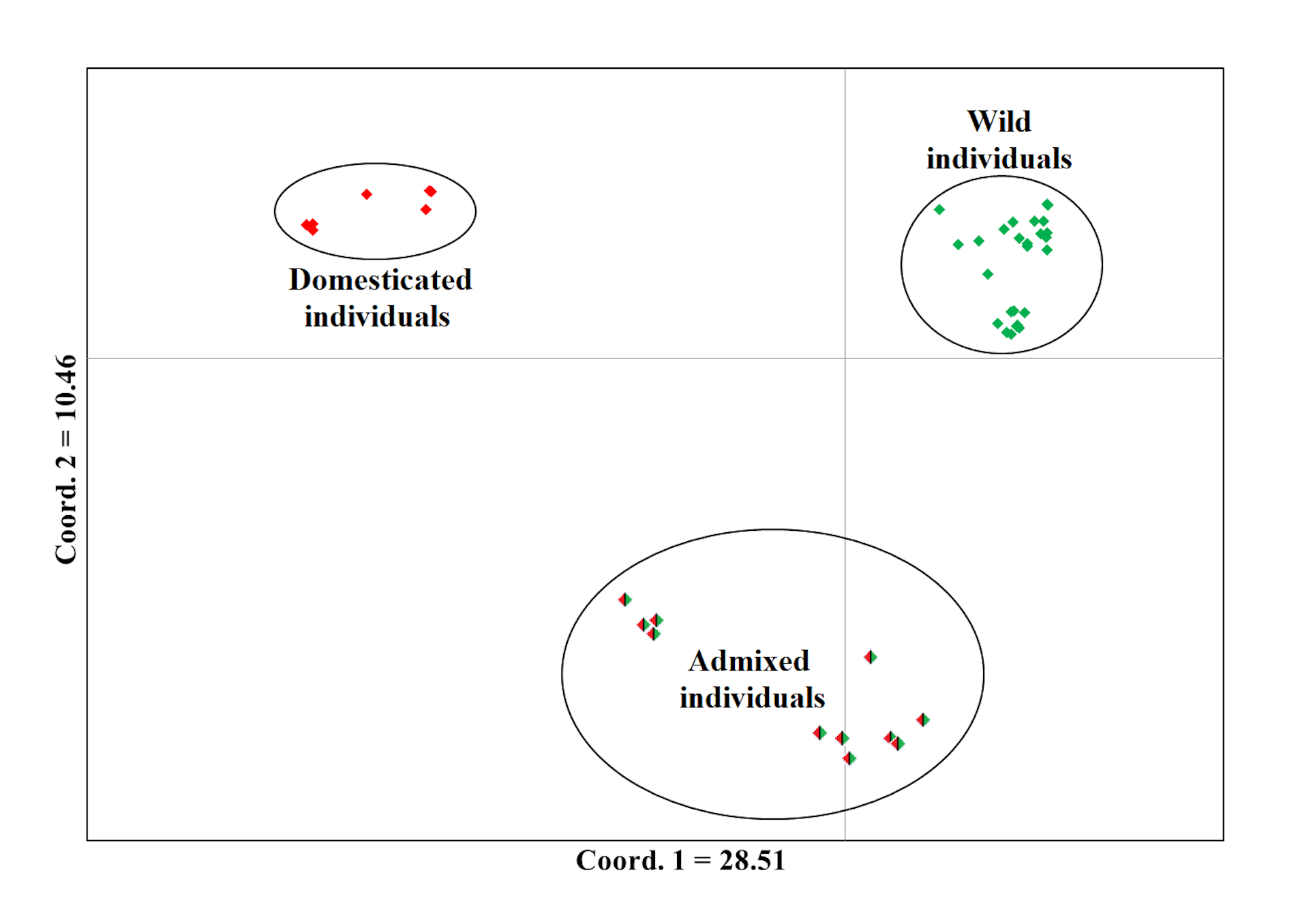

Supplement: Figure S2 — The colors assigned to the observed groups correspond to those used in the STRUCTURE analysis with K = 2 (Fig. 3-B). [file peerj-10-13690-s002.png]

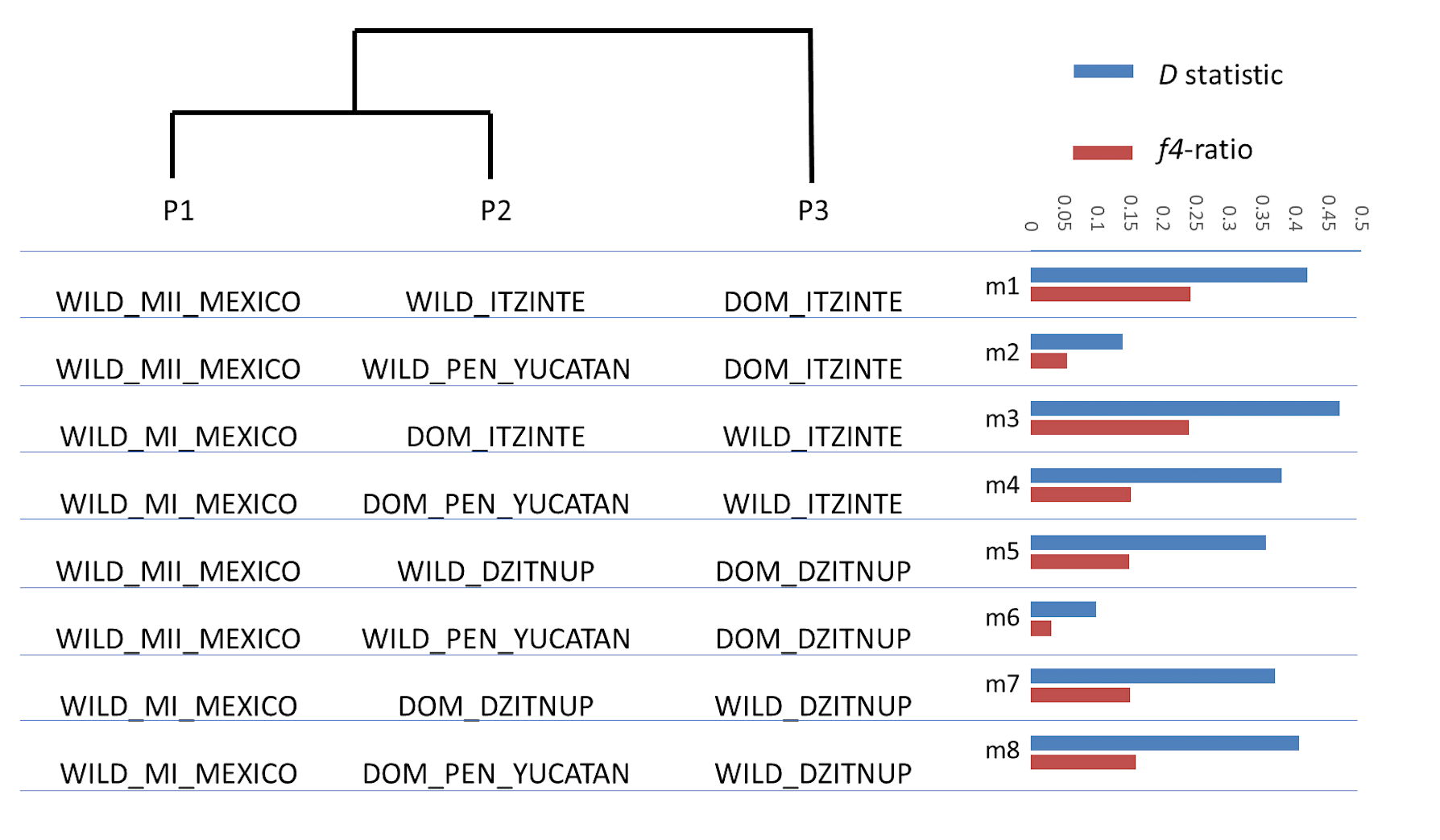

Supplement: Figure S3 — In these models, wild MII populations from Mexico (outside the Yucatan Peninsula) were assigned as the sister group (P1) of wild populations within the complexes or within the Yucatan Peninsula assigned as P2, and wild MI populations from Mexico were assigned as the sister group (P1) of domesticated populations within the complexes or within the Yucatan Peninsula assigned as P2. In each model P3, the tested group, was appropriately chosen to test gene flow between P3 and P2. In all these models, the Andean wild gene pool (AI) was fixed as outgroup. [file peerj-10-13690-s003.png]

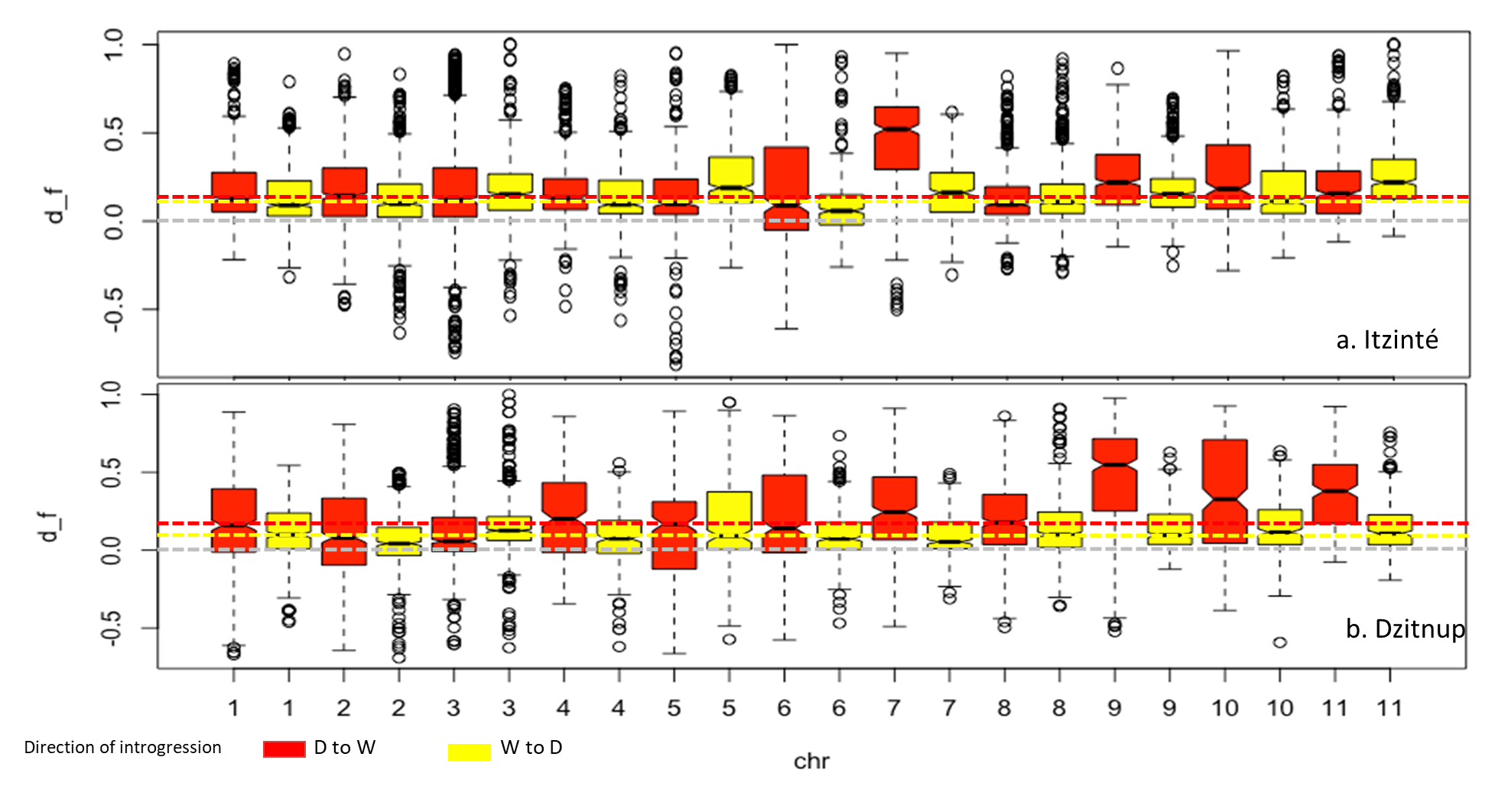

Supplement: Figure S4 — The figure shows the proportion of introgression (df) per chromosome in the Itzinté (a) and Dzitnup (b) complexes in the direction domesticated to wild (d-w) (red bars) and wild to domesticated (w-d) (yellow bars). Gray dashed lines indicate df = 0, red and yellow dashed lines indicate median df values observed in the direction d-w and w-d, respectively. [file peerj-10-13690-s004.png]

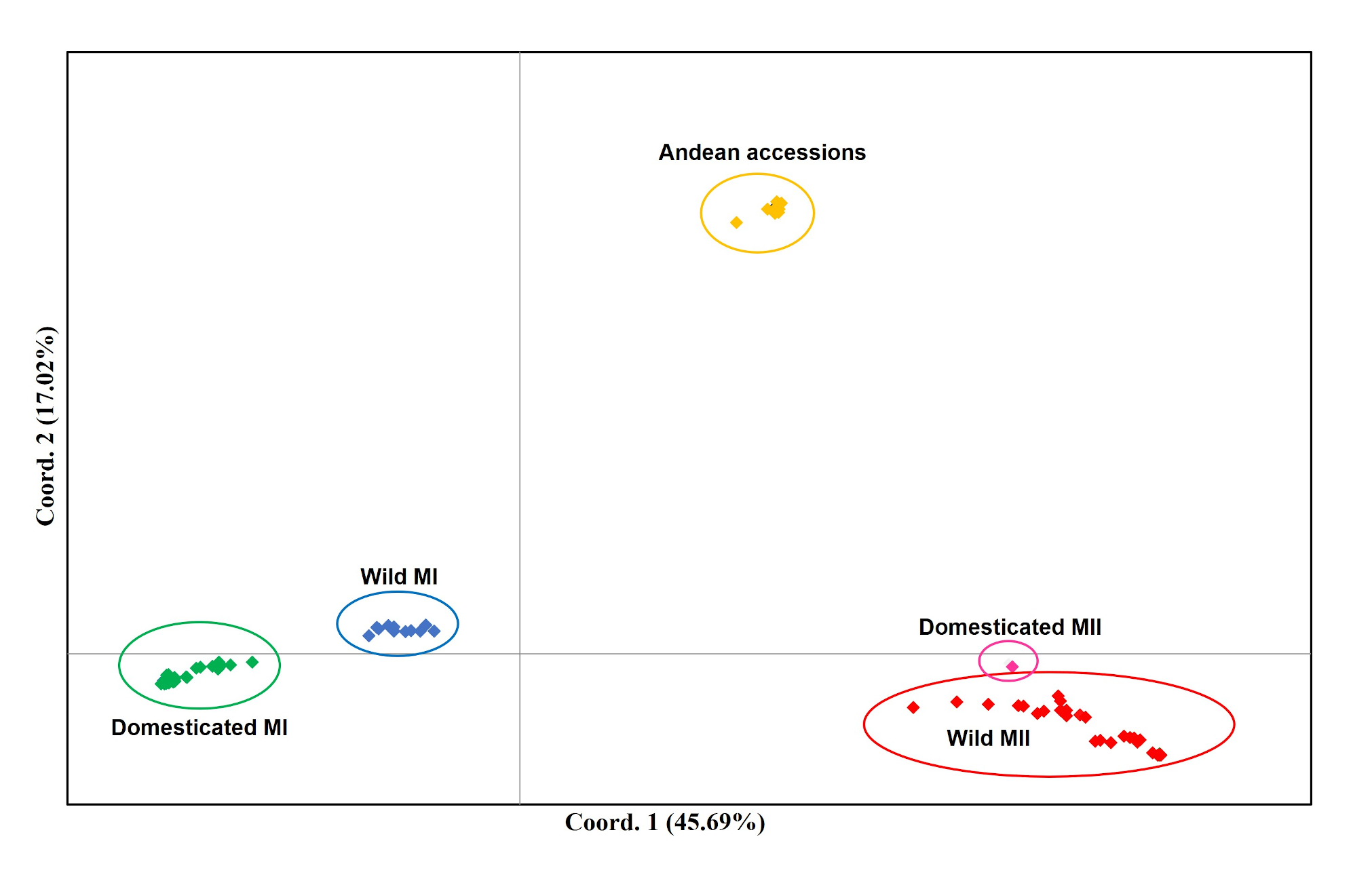

Supplement: Figure S5 — The colors assigned to the observed groups correspond to those used in the Neighbor-Joining analysis (Fig. 6). [file peerj-10-13690-s005.png]

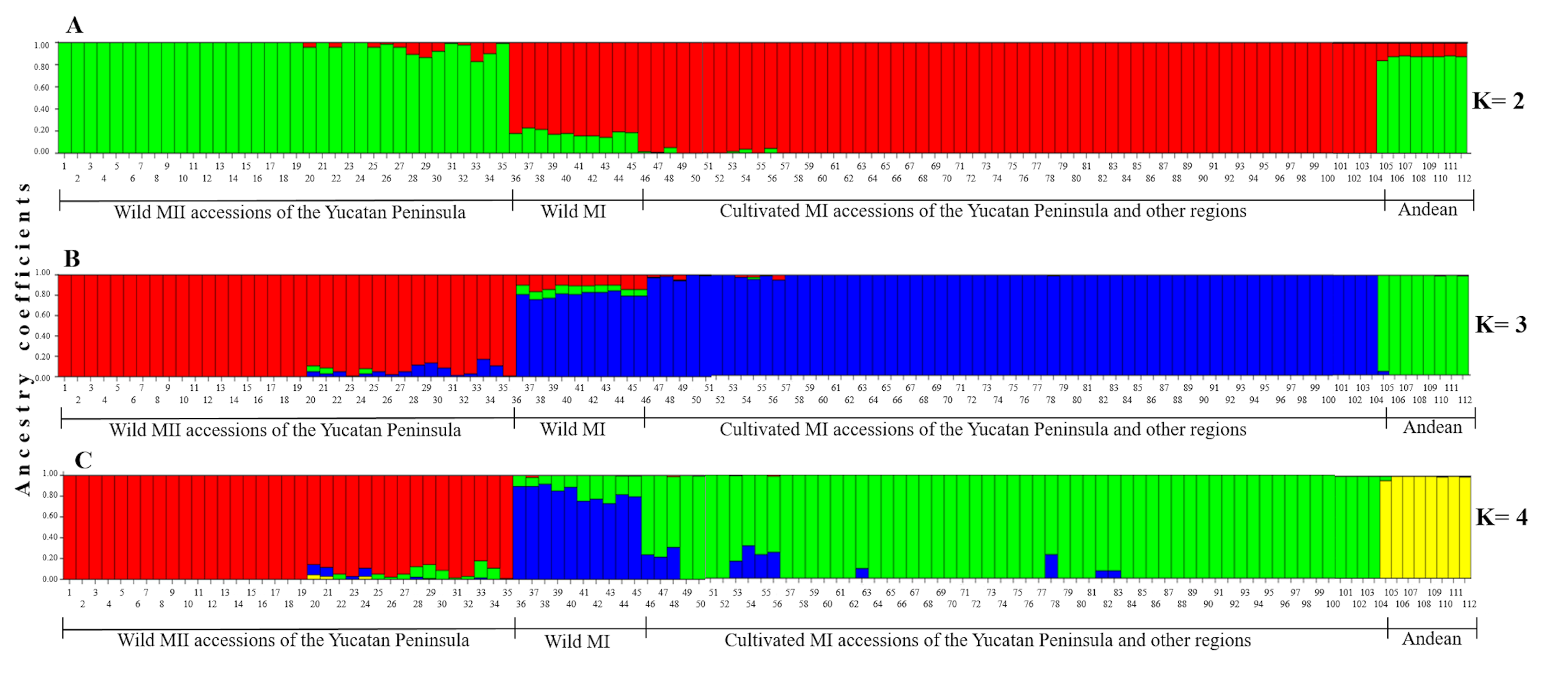

Supplement: Figure S6 — The colors correspond to the observed groups using different K values: (A) K = 2; (B) K = 3; (C) K = 4. Colors with K = 4 match to those used in the Neighbor-Joining analysis (Fig. 6) and in the PCoA (Fig. S5). [file peerj-10-13690-s006.png]

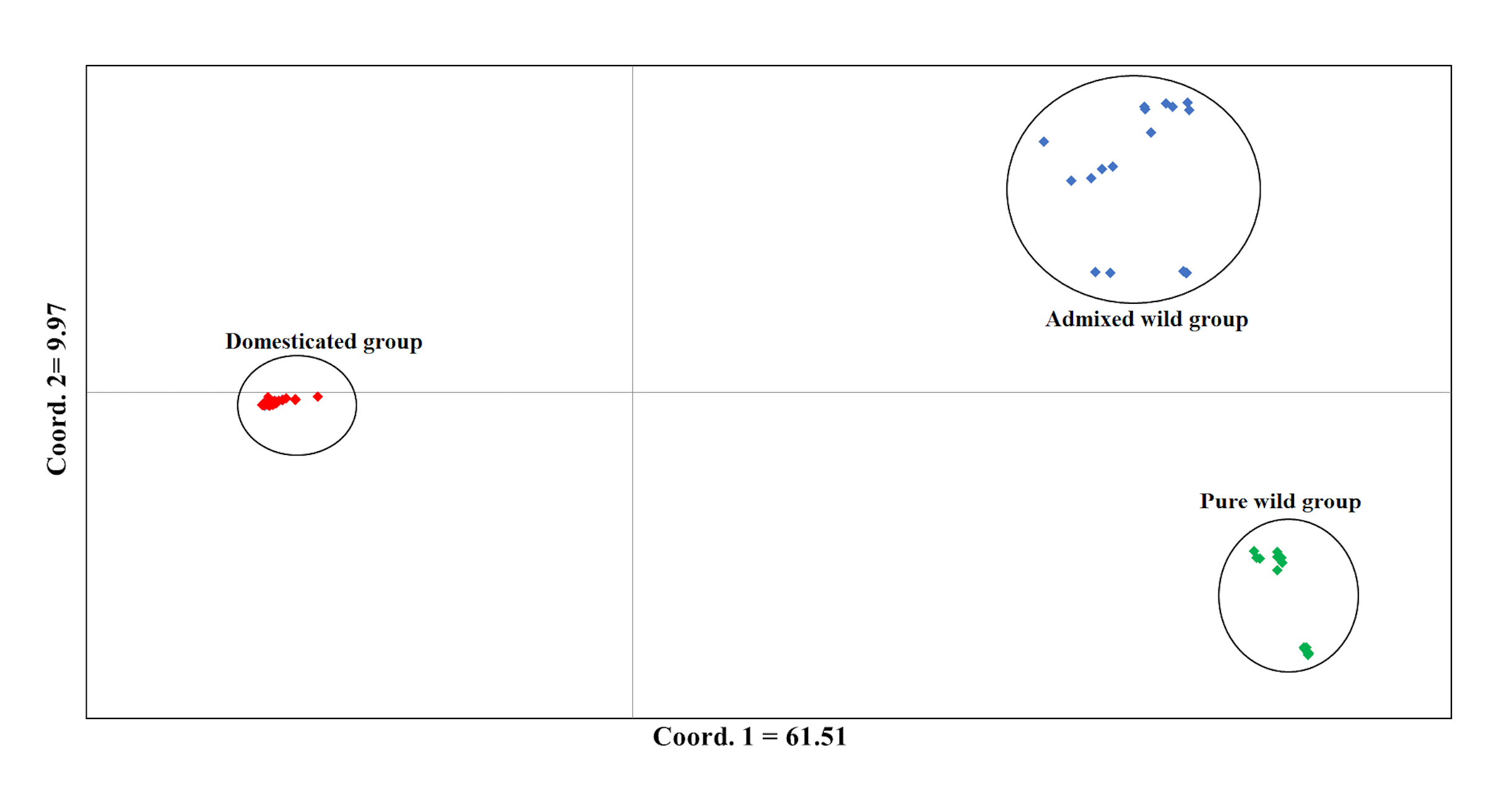

Supplement: Figure S7 — The colors assigned to the observed groups correspond to those used in the STRUCTURE analysis with K = 3 (Fig. 7). [file peerj-10-13690-s007.png]
