# Supplementary material for: Consequences of introgression and gene flow on the genetic structure and diversity of Lima bean (Phaseolus lunatus L.) in its Mesoamerican diversity area
Source: PeerJ. 2022 Jul 5;10:e13690. doi: 10.7717/peerj.13690 (PMC9266586; doi:10.7717/peerj.13690)
Supplement: Table S2 [file peerj-10-13690-s009.docx]

| **Supplementary material Table S2. Geographic location of the wild and domesticated accessions of Lima bean (*Phaseolus lunatus* L.) from de Yucatan Peninsula and the external group, used for the regional analysis.** | | | | | | |
| --- | --- | --- | --- | --- | --- | --- |
| **Accession ID** | **Gene pool** | **Biological status** | **Country** | **Province** | **Latitude** | **Longitude** |
| 8KM_282 | Wild_MII | Wild | Mexico | Tabasco | N 18° 35' 31'' | W 92° 33' 88'' |
| 8KM_283 | Wild_MII | Wild | Mexico | Tabasco | N 18° 35' 31'' | W 92° 33' 88'' |
| 8KM_285 | Wild_MII | Wild | Mexico | Tabasco | N 18° 35' 31'' | W 92° 33' 88'' |
| 8KM_288 | Wild_MII | Wild | Mexico | Tabasco | N 18° 35' 31'' | W 92° 33' 88'' |
| ATASTA_299 | Wild_MII | Wild | Mexico | Campeche | N 18° 37' 05'' | W 92° 09' 95'' |
| ATASTA_377 | Wild_MII | Wild | Mexico | Campeche | N 18° 37' 05'' | W 92° 09' 95'' |
| ATASTB_303 | Wild_MII | Wild | Mexico | Campeche | N 18° 37' 05'' | W 92° 09' 95'' |
| ATASTB_304 | Wild_MII | Wild | Mexico | Campeche | N 18° 37' 05'' | W 92° 09' 95'' |
| ATASTB_305 | Wild_MII | Wild | Mexico | Campeche | N 18° 37' 05'' | W 92° 09' 95'' |
| ATASTB_307 | Wild_MII | Wild | Mexico | Campeche | N 18° 37' 05'' | W 92° 09' 95'' |
| FNTA_272 | Wild_MII | Wild | Mexico | Tabasco | N 18° 32' 46'' | W 92° 38' 10'' |
| FNTA_273 | Wild_MII | Wild | Mexico | Tabasco | N 18° 32' 46'' | W 92° 38' 10'' |
| FNTB_276 | Wild_MII | Wild | Mexico | Tabasco | N 18° 32' 46'' | W 92° 38' 10'' |
| FNTB_278 | Wild_MII | Wild | Mexico | Tabasco | N 18° 32' 46'' | W 92° 38' 10'' |
| FNTB_281 | Wild_MII | Wild | Mexico | Tabasco | N 18° 32' 46'' | W 92° 38' 10'' |
| SMAPAC_289 | Wild_MII | Wild | Mexico | Campeche | N 18° 53' 58'' | W 91° 02' 62'' |
| SMAPAC_290 | Wild_MII | Wild | Mexico | Campeche | N 18° 53' 58'' | W 91° 02' 62'' |
| SMAPAC_291 | Wild_MII | Wild | Mexico | Campeche | N 18° 53' 58'' | W 91° 02' 62'' |
| G25750-COR | Wild_MII | Wild | Mexico | Campeche | N 20° 24' 36'' | W 90° 14' 23'' |
| G25785-COR | Wild_MII | Wild | Mexico | Campeche | N 20° 03' 00'' | W 89° 44' 23'' |
| G25816-ELS | Wild_MII | Wild | Mexico | Yucatan | N 20° 56' 60'' | W 89° 01' 48'' |
| JMC_1010 | Wild_MII | Wild | Mexico | Yucatan | N 20° 12' 11'' | W 89° 17' 16'' |
| JMC_1012 | Wild_MII | Wild | Mexico | Yucatan | N 20° 12' 11'' | W 89° 17' 16'' |
| JMC_1015 | Wild_MII | Wild | Mexico | Yucatan | N 20° 23' 43'' | W 89° 32' 20'' |
| JMC_1417 | Wild_MII | Wild | Mexico | Quintana Roo | N 19° 25' 60'' | W 88° 08' 30'' |
| JMC_1418 | Wild_MII | Wild | Mexico | Quintana Roo | N 19° 23' 48'' | W 88° 08' 10'' |
| JMC_221 | Wild_MII | Wild | Mexico | Yucatan | N 20° 31' 30'' | W 88° 07' 23'' |
| JMC_549 | Wild_MII | Wild | Mexico | Yucatan | N 20° 12' 38'' | W 89° 22' 08'' |
| JMC_609 | Wild_MII | Wild | Mexico | Yucatan | N 20° 12' 32'' | W 89° 22' 08'' |
| JMC_715 | Wild_MII | Wild | Mexico | Campeche | N 20° 13' 01'' | W 89° 43' 40'' |
| JMC_778 | Wild_MII | Wild | Mexico | Campeche | N 19° 24' 51'' | W 89° 34' 27'' |
| JMC_922 | Wild_MII | Wild | Mexico | Campeche | N 19° 56' 51'' | W 89° 44' 03'' |
| G25735-ELS | Domesticated_MI | Domesticated | Mexico | Campeche | N 20° 14' 24'' | W 89° 56' 23'' |
| G25766-ELS | Domesticated_MI | Domesticated | Mexico | Campeche | N 20° 08' 24'' | W 90° 13' 11'' |
| G25771-ELS | Domesticated_MI | Domesticated | Mexico | Campeche | N 20° 11' 60'' | W 89° 54' 35'' |
| G25787-ELS | Domesticated_MI | Domesticated | Mexico | Campeche | N 19° 35' 24'' | W 89° 36' 35'' |
| G26173-ELS | Domesticated_MI | Domesticated | Mexico | Yucatan | No data | No data |
| JMC_1003 | Domesticated_MI | Domesticated | Mexico | Yucatan | N 20° 23' 43'' | W 89° 32' 02'' |
| JMC_1005 | Domesticated_MI | Domesticated | Mexico | Yucatan | N 20° 12' 11'' | W 89° 17' 16'' |
| JMC_1007 | Domesticated_MI | Domesticated | Mexico | Yucatan | N 20° 12' 11'' | W 89° 17' 16'' |
| JMC_1024 | Domesticated_MI | Domesticated | Mexico | Campeche | N 20° 00' 12'' | W 89° 44' 52'' |
| JMC_1035 | Domesticated_MI | Domesticated | Mexico | Campeche | N 20° 04' 59'' | W 89° 49' 00'' |
| JMC_1059 | Domesticated_MI | Domesticated | Mexico | Quintana Roo | N 19° 51' 30'' | W 88° 10' 29'' |
| JMC_111 | Domesticated_MI | Domesticated | Mexico | Quintana Roo | N 20° 21' 32'' | W 88° 02' 11'' |
| JMC_112 | Domesticated_MI | Domesticated | Mexico | Yucatan | N 20° 35' 56'' | W 88° 09' 48' |
| JMC_1179 | Domesticated_MI | Domesticated | Mexico | Quintana Roo | N 19° 52' 45'' | W 88° 19' 00'' |
| JMC_1186 | Domesticated_MI | Domesticated | Mexico | Quintana Roo | N 19° 52' 45'' | W 88° 19' 00'' |
| JMC_1196 | Domesticated_MI | Domesticated | Mexico | Quintana Roo | N 19° 52' 45'' | W 88° 19' 00'' |
| JMC_1199 | Domesticated_MI | Domesticated | Mexico | Yucatan | N 20° 22' 09'' | W 88° 05' 11'' |
| JMC_1200 | Domesticated_MI | Domesticated | Mexico | Quintana Roo | N 19° 46' 30'' | W 88° 10' 59'' |
| JMC_1201 | Domesticated_MI | Domesticated | Mexico | Quintana Roo | N 21° 06' 10'' | W 87° 20' 16'' |
| JMC_1215 | Domesticated_MI | Domesticated | Mexico | Yucatan | N 20° 20' 58'' | W 89° 01' 09'' |
| JMC_1265 | Domesticated_MI | Domesticated | Mexico | Quintana Roo | N 19° 50' 35'' | W 88° 08' 03'' |
| JMC_1284 | Domesticated_MI | Domesticated | Mexico | Campeche | N 19° 34' 59'' | W 89° 34' 59'' |
| JMC_1288 | Domesticated_MI | Domesticated | Mexico | Yucatan | N 19° 58' 42'' | W 89° 29' 17'' |
| JMC_13 | Domesticated_MI | Domesticated | Mexico | Quintana Roo | N 19° 29' 39'' | W 87° 59' 39'' |
| JMC_1325 | Domesticated_MI | Domesticated | Mexico | Yucatan | N 20° 12' 11'' | W 89° 17' 16'' |
| JMC_277 | Domesticated_MI | Domesticated | Mexico | Yucatán | N 20° 19' 45'' | W 88° 11' 05'' |
| JMC_9 | Domesticated_MI | Domesticated | Mexico | Quintana Roo | N 19° 29' 39'' | W 89° 59' 39'' |
| JMC_995 | Domesticated_MI | Domesticated | Mexico | Yucatan | N 20° 53' 27'' | W 88° 08' 11'' |
| ARB_369 | Domesticated_MI | Domesticated | Mexico | Yucatan | No data | No data |
| ARB_371 | Domesticated_MI | Domesticated | Mexico | Yucatan | No data | No data |
| BGB_I | Domesticated_MI | Domesticated | Mexico | Yucatan | N 19° 52' 27'' | W 89° 13' 00'' |
| BGB_IV | Domesticated_MI | Domesticated | Mexico | Quintana Roo | N 19° 54' 55'' | W 89° 09' 34'' |
| BGB_IX | Domesticated_MI | Domesticated | Mexico | Quintana Roo | N 20° 48' 15'' | W 87° 28' 33'' |
| BGB_V | Domesticated_MI | Domesticated | Mexico | Quintana Roo | N 19° 39' 45'' | W 88° 26' 39'' |
| BGB_VI | Domesticated_MI | Domesticated | Mexico | Quintana Roo | N 19° 23' 30'' | W 88° 04' 27'' |
| BGB_VII | Domesticated_MI | Domesticated | Mexico | Quintana Roo | N 19° 52' 45'' | W 88° 19' 00'' |
| BGP_4 | Domesticated_MI | Domesticated | Mexico | Yucatan | N 20° 19' 27'' | W 89° 08' 01'' |
| BGP_5 | Domesticated_MI | Domesticated | Mexico | Quintana Roo | N 19° 54' 55'' | W 89° 09' 34'' |
| BGP_6 | Domesticated_MI | Domesticated | Mexico | Yucatan | N 20° 05' 53'' | W 89° 27' 46'' |
| BGP_7 | Domesticated_MI | Domesticated | Mexico | Yucatan | N 21° 22' 23'' | W 89° 00' 54'' |
| BGP_8 | Domesticated_MI | Domesticated | Mexico | Yucatan | N 20° 20' 03'' | W 88° 05' 17'' |
| BGP_9 | Domesticated_MI | Domesticated | Mexico | Yucatan | N 21° 00' 38'' | W 88° 18' 19'' |
| BGS_1 | Domesticated_MI | Domesticated | Mexico | Yucatan | N 19° 52' 27'' | W 89° 13' 00'' |
| BGS_2 | Domesticated_MI | Domesticated | Mexico | Yucatan | N 19° 46' 41'' | W 89° 02' 20'' |
| BGS_3 | Domesticated_MI | Domesticated | Mexico | Yucatan | N 21° 02' 06'' | W 89° 38' 08'' |
| BGS_4 | Domesticated_MI | Domesticated | Mexico | Campeche | N 20° 00' 21'' | W 89° 44' 58'' |
| BGS_5 | Domesticated_MI | Domesticated | Mexico | Quintana Roo | N 19° 54' 55'' | W 89° 09' 34'' |
| BGS_6 | Domesticated_MI | Domesticated | Mexico | Yucatan | N 20° 05' 53'' | W 89° 27' 46'' |
| BGS_7 | Domesticated_MI | Domesticated | Mexico | Yucatan | N 21° 22' 23'' | W 89° 00' 54'' |
| BGS_8 | Domesticated_MI | Domesticated | Mexico | Yucatan | N 20° 18' 55'' | W 89° 01' 39'' |
| BGS_9 | Domesticated_MI | Domesticated | Mexico | Yucatan | N 19° 04' 35'' | W 88° 27' 23'' |
| JMC_1377 | Domesticated_MI | Domesticated | Mexico | Chiapas | N 16° 45' 34'' | W 92° 43' 17'' |
| G25229-ELS | Wild_MI | Wild | Mexico | Nayarit | N 20° 49' 59'' | W 105° 24' 00'' |
| G25230-ELS | Wild_MI | Wild | Mexico | Colima | N 19° 03' 00'' | W 104° 13' 59'' |
| G25231-COR | Wild_MI | Wild | Mexico | Colima | N 19° 01' 59'' | W 104° 13' 00'' |
| G26358-COR | Wild_MI | Wild | Mexico | Morelos | N 18° 53' 60'' | W 98° 58' 59'' |
| G26360-ELS | Wild_MI | Wild | Mexico | Puebla | N 18° 43' 59'' | W 98° 27' 00'' |
| G26517-ELS | Wild_MI | Wild | Mexico | Guerrero | N 16° 40' 00'' | W 98° 27' 00'' |
| JMC_1074 | Wild_MI | Wild | Mexico | Oaxaca | N 15° 57' 26'' | W 97° 21' 51'' |
| JMC_1075 | Wild_MI | Wild | Mexico | Oaxaca | N 16° 01' 17'' | W 97° 33' 03'' |
| G25614-ELS | Domesticated_MI | Domesticated | Mexico | Oaxaca | N 17° 18' 00'' | W 96° 54' 00'' |
| G26512-ELS | Domesticated_MI | Domesticated | Mexico | Guerrero | N 18° 10' 00'' | W 100° 28' 59'' |
| G26534-ELS | Domesticated_MI | Domesticated | Mexico | Chiapas | N 16° 19' 59'' | W 92° 25' 59'' |
| G27399-ELS | Domesticated_MI | Domesticated | Mexico | Veracruz | N 20° 28' 59'' | W 97° 27' 00'' |
| JMC_1105 | Domesticated_MI | Domesticated | Guatemala | Petén | N 16° 59' 38'' | W 89° 55' 03'' |
| G25970-COR | Wild_MII | Wild | Mexico | Morelos | N 18° 53' 60'' | W 99° 01' 59'' |
| G27578-COR | Wild_MII | Wild | Guatemala | Chimaltenango | N 14° 52' 59'' | W 90° 57' 00'' |
| G25172-ELS | Domesticated_Andean | Domesticated | Peru | Ica | S 13° 42' 36'' | W 76° 13' 11'' |
| G25400-ELS | Domesticated_Andean | Domesticated | Colombia | Cauca | N 02° 07' 12'' | W 76° 57' 35'' |
| G25420-ELS | Domesticated_Andean | Domesticated | Peru | Piura | S 05° 11' 20'' | W 80° 39' 41'' |
| G25910-ELS | Domesticated_Andean | Domesticated | Colombia | Nariño | N 01° 15' 00'' | W 77° 40' 00'' |
| G26348-ELS | Wild_Andean | Wild | Peru | Cajamarca | S 06° 20' 60'' | W 79° 24' 00'' |
| G26459-ELS | Wild_Andean | Wild | Ecuador | Loja | S 03° 56' 24" | W 79° 24' 36" |
| G26480-ELS | Domesticated_Andean | Domesticated | Ecuador | Imbabura | N 00° 24' 00'' | W 78° 07' 59'' |
| G26609-ELS | Wild_Andean | Wild | Ecuador | Azuay | S 03° 13' 00'' | W 79° 12' 00'' |
| G25254-ELS | Domesticated_MI | Domesticated | Guatemala | Suchitepequez | N 14° 32' 24'' | W 91° 30' 00'' |
| G25267-COR | Domesticated_MI | Domesticated | El Salvador | San Salvador | N 13° 41' 60'' | W 89° 13' 00'' |
| G25388-COR | Wild_MII | Wild | Costa Rica | San Jose | N 9° 59 '24" | W 84° 04 '12" |

| **Supplementary material Table S5.** Models used for the ABBA-BABA tests. Populations: WILD_MII_MEXICO: MII wild populations from Mexico and outside the Yucatan Peninsula. WILD_ITZINTE: Wild populations collected within the Itzinte complex. DOM_ITZINTE: domesticated populations collected within the Itzinte complex. WILD_DZITNUP: Wild populations collected within the Dzitnup complex. DOM_DZITNUP: domesticated populations collected within the Dzitnup complex. WILD_PEN_YUCATAN: Wild MII populations from the Yucatan Peninsula, outside the two complexes. | | | | | | | | | | |
| --- | --- | --- | --- | --- | --- | --- | --- | --- | --- | --- |
| **Model** | **P1** | **P2** | **P3** | ***D* statistic** | **Z-score** | **p-value** | ***f4*-ratio** | **BBAA** | **ABBA** | **BABA** |
| m1 | WILD_MII_MEXICO | WILD_ITZINTE | DOM_ITZINTE | 0.417901 | 16.115 | 0 | 0.24124 | 1060.14 | 588.344 | 241.536 |
| m2 | WILD_MII_MEXICO | WILD_PEN_YUCATAN | DOM_ITZINTE | 0.138807 | 4.86032 | 1.17E-06 | 0.054742 | 1500.5 | 322.769 | 244.086 |
| m3 | WILD_MI_MEXICO | DOM_ITZINTE | WILD_ITZINTE | 0.467087 | 16.2133 | 0 | 0.239214 | 989.261 | 482.265 | 175.181 |
| m4 | WILD_MI_MEXICO | DOM_PEN_YUCATAN | WILD_ITZINTE | 0.380198 | 11.3697 | 0 | 0.150944 | 1067.21 | 351.713 | 157.943 |
| m5 | WILD_MII_MEXICO | WILD_DZITNUP | DOM_DZITNUP | 0.355316 | 9.56638 | 0 | 0.149456 | 1254.48 | 494.053 | 235.006 |
| m6 | WILD_MII_MEXICO | WILD_PEN_YUCATAN | DOM_DZITNUP | 0.0980746 | 3.4773 | 0.0005065 | 0.0303635 | 1570.36 | 294.621 | 241.993 |
| m7 | WILD_MI_MEXICO | DOM_DZITNUP | WILD_DZITNUP | 0.369491 | 11.2768 | 0 | 0.149572 | 1095.66 | 352.012 | 162.065 |
| m8 | WILD_MI_MEXICO | DOM_PEN_YUCATAN | WILD_DZITNUP | 0.405745 | 11.3638 | 0 | 0.159135 | 1099.97 | 350.122 | 148.008 |

| **Supplementary material Table S6.** D and df values evaluating introgression between wild and domesticated populations within the complexes Itzinté and Dzitnup for models m1, m3, m5 and m7 (see Supplementary material 4). | | | | | | | | |
| --- | --- | --- | --- | --- | --- | --- | --- | --- |
| Gene flow direction | domesticated-wild | | wild-domesticated | | domesticated-wild | | wild-domesticated | |
| model | m1 | | m3 | | m5 | | m7 | |
| Complex | Itzinté | | | | Dzitnup | | | |
| Chromosome | *D* | *df* | *D* | *df* | *D* | *df* | *D* | *df* |
| 1 | 0.441457 | 0.123901 | 0.502403 | 0.091091 | 0.2893145 | 0.1603445 | 0.488253 | 0.099038 |
| 2 | 0.3970185 | 0.1418055 | 0.481165 | 0.099166 | 0.141952 | 0.07882 | 0.271332 | 0.042128 |
| 3 | 0.3696465 | 0.119632 | 0.642111 | 0.151318 | 0.225897 | 0.0565745 | 0.6058775 | 0.125438 |
| 4 | 0.498131 | 0.12997 | 0.42828 | 0.094898 | 0.354216 | 0.200832 | 0.278843 | 0.074157 |
| 5 | 0.386699 | 0.0984715 | 0.623118 | 0.188345 | 0.2541365 | 0.1674475 | 0.44466 | 0.090465 |
| 6 | 0.22791 | 0.089688 | 0.2843225 | 0.057733 | 0.328636 | 0.140399 | 0.352713 | 0.0730415 |
| 7 | 0.5853025 | 0.5205015 | 0.531635 | 0.1609345 | 0.33729 | 0.244697 | 0.405934 | 0.0534455 |
| 8 | 0.414284 | 0.09456 | 0.54878 | 0.107578 | 0.330045 | 0.17705 | 0.477634 | 0.100241 |
| 9 | 0.519456 | 0.219054 | 0.535529 | 0.152653 | 0.681865 | 0.547782 | 0.502901 | 0.096698 |
| 10 | 0.52679 | 0.182697 | 0.5237175 | 0.1119735 | 0.474535 | 0.326799 | 0.40526 | 0.114531 |
| 11 | 0.493993 | 0.1552535 | 0.628684 | 0.219698 | 0.5119445 | 0.378821 | 0.394584 | 0.1069955 |
| Median | 0.441457 | 0.12997 | 0.531635 | 0.1119735 | 0.330045 | 0.17705 | 0.405934 | 0.096698 |

| **Supplementary material Table S7.** Size of introgressed blocks per chromosome between wild and domesticated accessions within each complex, for the 10% top values of df within each chromosome. Direction of introgression: d_w: domesticated to wild, w_d: wild to domesticated. | | | | | |
| --- | --- | --- | --- | --- | --- |
| Chromosome | Dzitnup d_w | Dzitnup_w_d | Itzinté d_w | Itzinté w_d | chr_length |
| 1 | 4187624 | 2737319 | 4938443 | 7519203 | 47515051 |
| 2 | 7345859 | 3845838 | 11460022 | 4248603 | 51573859 |
| 3 | 10488417 | 15142349 | 10038459 | 12384642 | 44992856 |
| 4 | 6283052 | 4425219 | 5298725 | 17835184 | 49404888 |
| 5 | 6975343 | 3627665 | 5523599 | 18774731 | 38037901 |
| 6 | 4459683 | 6266291 | 6161261 | 6620601 | 36496517 |
| 7 | 7873610 | 9039978 | 5250650 | 7806577 | 47783237 |
| 8 | 9980349 | 8420044 | 11060978 | 24998671 | 57224906 |
| 9 | 16231923 | 7934419 | 9035578 | 11404792 | 40827468 |
| 10 | 5872552 | 7166399 | 2981684 | 4633031 | 53892040 |
| 11 | 7914568 | 4355050 | 4165001 | 10677411 | 48004042 |
| Total length (bp) | 87.612.980 | 70.223.252 | 75.914.400 | 119.384.243 | 515.752.765 |
| # of introgressed blocks | 64 | 65 | 90 | 99 |  |
| Average size of block in bp | 1.368.953 | 1.080.357 | 843.493 | 1.205.901 |  |

| **Supplementary material Table S8**. Analysis of molecular variance (AMOVA) of wild and domesticated accessions of Lima bean from the Yucatan Peninsula, using 15,169 SNP markers. | | | | |
| --- | --- | --- | --- | --- |
| Source of variation | d. f. | Sum of squares | Variance components | Percentage of variation |
| Among groups (wild and domesticated) | 2 | 78996.961 | 862.11661 Va | 81.74 |
| Among individuals  within groups | 80 | 23851.388 | 105.59527 Vb | 10.01 |
| Within individuals | 83 | 7217.000 | 86.95181 Vc | 8.24 |
| Total | 165 | 110065.349 | 1054.66369 | 100 |
| Fixation Indices  *F*_IS_: 0.54841  *F*_ST_: 0.81743  *F*_IT_: 0.91755 |  |  |  |  |
