# Supplementary material for: Consequences of introgression and gene flow on the genetic structure and diversity of Lima bean (Phaseolus lunatus L.) in its Mesoamerican diversity area
Source: PeerJ. 2022 Jul 5;10:e13690. doi: 10.7717/peerj.13690 (PMC9266586; doi:10.7717/peerj.13690)
Supplement: Table S4 [file peerj-10-13690-s011.docx]

| Supplemental Material Table S4. NGSEP results |
| --- |
|  |
