# Supplementary material for: Consequences of introgression and gene flow on the genetic structure and diversity of Lima bean (Phaseolus lunatus L.) in its Mesoamerican diversity area
Source: PeerJ. 2022 Jul 5;10:e13690. doi: 10.7717/peerj.13690 (PMC9266586; doi:10.7717/peerj.13690)
Supplement: Table S6 [file peerj-10-13690-s012.docx]

***Supplementary Table S6.1*.** Chromosomal regions with evidence of introgression and location of domestication QTL.

| Trait | QTL peak* | Dzitnup_d_w | Dzitnup_w_d | Itzinté_d_w | Itzinté_w_d |
| --- | --- | --- | --- | --- | --- |
| Determinacy | Pl01_42432553 | Yes | no | yes | no |
| Hundred-seed weight (g) | Pl10_39674709 | no | no | no | no |
|  | Pl03_26637359 | no | no | yes | no |
|  | Pl09_6453659 | no | no | no | yes |
|  | Pl04_2884774 | no | no | no | yes |
| Flowering time | Pl01_42432553 | yes | no | yes | no |
| Cyanogenesis | Pl05_35651614 | no | no | no | no |
|  | Pl10_44773521 | no | no | no | No |
|  | Pl08_2715645 | yes | no | yes | No |
| Seed coat color | Pl07_46475134 | yes | yes | no | No |
|  | Pl07_45067785 | no | yes | no | Yes |

According to García et al. (2021)

In Lima bean, very little is known about the genetic control of the domestication syndrome. The study of Garcia et al. (2021) reported QTL for determinacy, hundred-seed weight, flowering time, cyanogenesis and seed coat color (see Supplementary table 4 of Garcia et al. 2021). In the table above we report the QTL that overlap with introgression blocks detected at Itzinté and Dzitnup in the present study (in the table “yes” mean that there is overlap between the location of the QTL and the location of introgression blocks detected at the complex, and “no” means that there is no overlap).

We can see that the QTL on chromosome 1, related to determinacy and flowering time, colocalize with an introgression block, in the direction domesticated to wild, observed in both complexes. Because in both complexes wild and domesticated materials are indeterminate, we think that these introgressions might not carry any phenotypic consequence for growth habit. However, this might not be the case for flowering time, a trait that might differ between wild and domesticated materials.

Regarding seed weight, we observed introgressions that colocalize with QTL related to this trait only at the Itzinté complex, in the direction wild to domesticated (QTLs on chromosomes 9 and 4) and in the direction domesticated to wild (QTL on chromosome 3). We think these introgressions might have phenotypic consequences on this trait given the notable difference that exist in seed size among wild and domesticated materials; this is a subject that deserves further investigation.

For cyanogenesis, we only observed an introgression block that colocalize with the QTL on chromosome 8 in the direction domesticated to wild in both complexes. Finally, for seed coat color, the first QTL on chromosome 7 (Pl07_46475134), colocalize with introgression blocks observed in both directions and only at Dzitnup. The second QTL (Pl07_45067785) colocalizes with an introgression block in the direction wild to domesticated observed at both complexes. In summary we can see that introgression blocks may include regions where QTL have been reported for several traits in Lima bean, however we should note that in the QTL experiment carried out by Garcia et al. (2021), the cross involved domesticated parents only, so it is uncertain whether these same QTL would be also significant for traits of the domestication syndrome in crosses that involve wild and domesticated materials.

***Supplementary Table S8*.** Number of introgression blocks unique and shared among the Dzitnup and Itzinté complexes.

| Chromosome | No. of introgression blocks | | | |
| --- | --- | --- | --- | --- |
|  | Shared among complexes | Unique to Dzitnup | Unique to Itzinté | Total |
| PL01 | 9 | 2 | 11 | 22 |
| PL02 | 10 | 5 | 17 | 32 |
| PL03 | 24 | 17 | 6 | 47 |
| PL04 | 8 | 3 | 12 | 23 |
| PL05 | 10 | 4 | 10 | 24 |
| PL06 | 10 | 6 | 5 | 21 |
| PL07 | 12 | 5 | 5 | 22 |
| PL08 | 18 | 8 | 11 | 37 |
| PL09 | 15 | 15 | 15 | 45 |
| PL10 | 7 | 1 | 4 | 12 |
| PL11 | 9 | 5 | 10 | 24 |
| Total | 132 (43%) | 71 (23%) | 106 (34%) | 309 |

A chi-square statistical test shows that the introgression blocks observed at the present study are not randomly distributed between chromosomes (chi-square= 50.514, p<0.0001). For example, in some chromosomes there are more introgression blocks than expected (in chromosomes PL02, PL03, PL05, PL08 and PL09). In figure 5 we have drawn the approximate location of the centromere in each chromosome and introgression blocks, in general, are located away from the centromere and are mostly located in euchromatic regions. Also, even though many introgression blocks are unique to one complex, there are many blocks that are shared among complexes. In the table above we can see that 43% of the total number of introgression blocks are shared among complexes, 23% are unique to Dzitnup and 34% are unique to Itzinté. So, we can conclude that the position of introgression blocks is highly correlated among complexes, which agrees with the observation above about the non-random distribution of these blocks in the genome of Lima bean. We still do not know if there are genomic features that may account for this interesting result and this is a subject that needs further research.
